# Reversible Constrained Dissociation and Reassembly of MXene Films

**DOI:** 10.1002/advs.202309171

**Published:** 2024-04-06

**Authors:** Xuefeng Zhang, Xudong Liu, Qingqiang Liu, Yufa Feng, Si Qiu, Ting Wang, Huayu Xu, Hao Li, Liang Yin, Hui Kang, Zhimin Fan

**Affiliations:** ^1^ School of chemistry and Materials Engineering Guangdong Provincial Key Laboratory for Electronic Functional Materials and Devices Huizhou University Huizhou 516007 China; ^2^ School of Materials Science and Engineering Harbin Institute of Technology Harbin 150001 China; ^3^ MIIT Key Laboratory of Critical Materials Technology for New Energy Conversion and Storage School of Chemistry and Chemical Engineering Harbin Institute of Technology Harbin 150001 China; ^4^ Advanced Materials Thrust The Hong Kong University of Science and Technology (Guangzhou) Guangzhou 510000 China

**Keywords:** complex structures, dissociation and reconstruction, Mxene film, reversible constraint, water‐induced

## Abstract

Enabling materials to undergo reversible dynamic transformations akin to the behaviors of living organisms represents a critical challenge in the field of material assembly. The pursuit of such capabilities using conventional materials has largely been met with limited success. Herein, the discovery of reversible constrained dissociation and reconfiguration in MXene films, offering an effective solution to overcome this obstacle is reported. Specifically, MXene films permit rapid intercalation of water molecules between their distinctive layers, resulting in a significant expansion and exhibiting confined dissociation within constrained spaces. Meanwhile, the process of capillary compression driven by water evaporation reinstates the dissociated MXene film to its original compact state. Further, the adhesive properties emerging from the confined disassociation of MXene films can spontaneously induce fusion between separate films. Utilizing this attribute, complex structures of MXene films can be effortlessly foamed and interlayer porosity precisely controlled, using only water as the inducer. Additionally, a parallel phenomenon has been identified in graphene oxide films. This work not only provides fresh insights into the microscopic mechanisms of 2D materials such as MXene but also paves a transformative path for their macroscopic assembly applications in the future.

## Graphical abstract

The unique reversible transformation ability and mechanism of hydrophilic 2D materials similar to the fission and aggregation of organisms is revealed. Harnessing this property allows the internal structure of 2D materials to be reconfigured, resulting in intricate porous structures solely through water infiltration. This discovery paves the way for revolutionary macro assembly applications for MXene.2

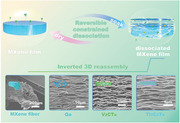

## 1 Introduction

The emergence and deployment of new materials are foundational to advancements in science and technology. 2D materials, spearheaded by graphene, have garnered significant interest due to their distinct topological structures, presenting extensive potential across various domains.^[^ ^1^, ^2^ ^]^ Recently, the biomimetic principle of “Taoism and Nature” has radically transformed the design and multifunctionality of 2D materials, endowing them with dynamic, life‐imitating structures and behaviors. For instance, graphene‐epoxy composites with a pearl‐layered structure demonstrate exceptional fracture toughness,^[^ ^3^ ^]^ while wet‐spun graphene fibers display behaviors that are evocative of cellular division and fusion.^[^ ^4^ ^]^ However, a predominant limitation is that the majority of 2D materials, including graphene, are prone to losing a considerable fraction of their inherent properties upon undergoing macroscopic assembly through conventional methods such as vacuum‐assisted filtration, evaporation, or spraying.^[^ ^5^, ^6^, ^7^ ^]^ Furthermore, once structured, these materials' internal micro‐architecture solidifies, diminishing the flexibility for future modifications or redesigns. Therefore, the challenge remains: how to imbue 2D macro assemblies with dynamic transformations akin to living organisms and to provide them with a ‘second life’ that enables the reconfiguration of their internal microstructures.

In 2011, the scientific community was acquainted with a groundbreaking 2D material known as MXenes. Over a decade, MXenes have emerged as the most comprehensive family of 2D materials.^[^ ^8^, ^9^ ^]^ MXenes predominantly consist of transition metal carbides, nitrides, and carbonitrides. They uniquely integrate metallic conductivity, high hydrophilicity, and exceptional redox activity—attributes not found in other known materials. For example, the Ti_3_C_2_T*_x_* MXene showcases impressive electrical conductivity, achieving up to 20 000 S cm^−1^, coupled with an ultra‐fast pseudocapacitive energy storage capability, a feature absent in conventional batteries and supercapacitors.^[^ ^10^, ^11^ ^]^ Consequently, MXenes have attracted considerable attention in pioneering fields, including seawater desalination,^[^ ^12^, ^13^ ^]^ electromagnetic interference shielding,^[^ ^14^, ^15^ ^]^ electrochemical energy storage,^[^ ^16^, ^17^ ^]^ tribology,^[^ ^18^, ^19^ ^]^ and electrocatalysis.^[^ ^20^ ^]^ A noteworthy attribute of MXenes is their pronounced sensitivity to water molecules. The surface terminal groups of MXenes not only enhance their hydrophilicity but also significantly reduce the diffusion resistance of water molecules.^[^ ^21^ ^]^ Furthermore, water molecules can selectively intercalate between distinct MXene nanolayers, thereby diminishing the interaction forces among these sheets.^[^ ^22^, ^23^, ^24^ ^]^ Drawing parallels from biology, single cells of slime molds, under arid conditions coalesce to form multicellular assemblies, undergoing metamorphosis and releasing spores through mitosis. Upon the return of humidity, these organisms revert to their reproductive and divisional processes (Figure S1, Supporting Information).^[^ ^25^ ^]^ Given the properties of MXenes, there exists potential for them to emulate dynamic recombination behaviors akin to slime molds. This presents a tantalizing prospect for the dynamic reconfiguration and controllable secondary assembly of MXene microstructures in aqueous environments—a challenge yet to be surmounted.

In this work, we have discovered a reversible constrained dissociation and reconfiguration phenomenon in MXene films for the first time. Through simple water wetting, the substantial hydration energy of the interlayer ions in MXene films instigates the spontaneous ingress of water molecules into the nanoscale interlayers, engendering low integer layers of orderly arrayed water molecules. This initiates a significant expansion due to the increased interlayer distance with disordered water molecules, culminating in the confined dissociation of the fundamental structural units of the nanosheets comprising the film. Moreover, the capillary compression induced by water evaporation can reciprocally transform the dissociated MXene into a densely packed film, demonstrating exceptional reversibility. Exploiting this phenomenon opens avenues for precise control over the microscopic pore structure and macroscopic morphology of MXene films. Such a discovery holds promise in broadening the horizons of film applications for 2D materials.

## 2 Results and Discussion

**Figure 1:**
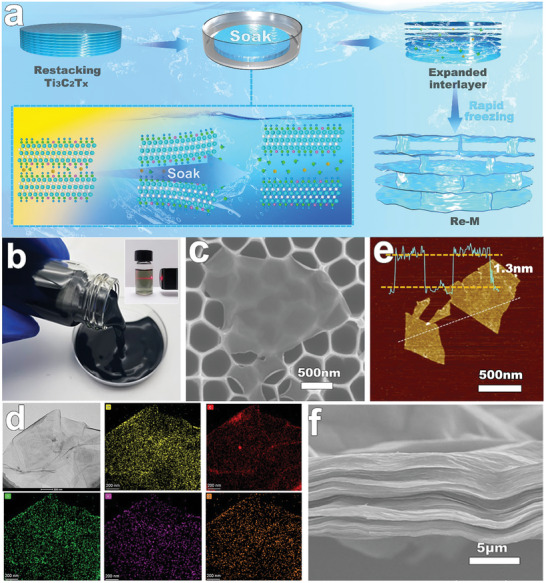
Reversible constrained dissociation and reassembly of MXene films. a) Schematic illustration of reversible constrained dissociation and 3D reassembly of MXene films. b) Photograph of a monolayer MXene (Ti_3_C_2_T*_x_*) dispersion, with an inset showing a Tyndall effect for diluted MXene dispersion. c) SEM image of MXene nanosheet. d) TEM image and element distribution of MXene nanosheet. e) AFM image and thickness of monolayer MXene nanosheet. f) Cross‐section SEM image of MXene film.

The reversible constrained dissociation and reassembly process of MXene films is schematically illustrated in **Figure** **1**a. Initially, a monolayer MXene (Ti_3_C_2_T*_x_*) dispersion was synthesized through an improved minimally intensive layer delamination method (Figure 1b). This technique is not only mature but also suitable for large‐scale production. Subsequently, through the application of vacuum‐assisted filtration of the MXene dispersion onto a cellulose filter membrane, the 2D topological MXene nanosheets are arranged and stacked to form free‐standing MXene films with a highly organized structure. Upon immersion of the MXene film in water, the hydration energy of the intercalating ions facilitates the rapid penetration of water molecules into specific interlayers of the nanosheets.^[^ ^22^, ^26^ ^]^ Within these constrained domains, predominantly low integer layers of water molecules are formed. As the interlayer spacing progressively expands, an electrostatic discrepancy between the hydrated ions and the nanosheet surfaces induces selective water molecule adsorption on both sides of the nanosheets. This phenomenon leads to the emergence of a diffuse double layer. Consequently, this process promotes further widening of the nanosheet layer spacing, ultimately resulting in the formation of a significantly swollen, disordered layer of water molecules between the sheets.^[^ ^27^ ^]^ This process propels the MXene film into a state of constrained dissociation. When these confined, dissociated MXene films are exposed to air, the longitudinal capillary compression force arising from water evaporation causes the films to revert to their original form.^[^ ^28^ ^]^ This showcases the reversible nature of the constrained dissociation and reconfiguration. Notably, in their dissociated state, the MXene films demonstrate adhesive characteristics, allowing multiple films to spontaneously merge into a single unit. This unified structure maintains the properties of reversible constrained dissociation and reconstruction.

To fully harness the potential of MXene, it's imperative to synthesize high‐quality MXene nanosheets. In pursuit of this, we conducted a comprehensive examination of the morphology and structure of the synthesized MXene. As shown in Figure 1b, the freshly prepared MXene dispersion manifests as black in color. Upon dilution, it transitions to a light green hue, accompanied by a pronounced Tyndall effect. Furthermore, the MXene film, assembled through filtration from the MXene dispersion, exhibited a lavender hue (Figure S2, Supporting Information). These distinct colorations are characteristic of Ti_3_C_2_T*_x_*, a specific MXene, providing preliminary evidence of successful MXene nanosheet synthesis.^[^ ^5^, ^14^ ^]^ Given the scanning electron microscope (SEM) ability to rapidly visualize the structure of 2D materials, we employed it to assess the microscopic morphology of the MXene nanosheets. It is evident that MXene exhibits typical 2D layered structural features (Figure 1c), markedly different from its precursor Ti_3_AlC_2_ (Figure S3, Supporting Information). Importantly, the surface of the MXene nanosheets is clean and unblemished, with virtually no presence of oxidized particles. Transmission electron microscopy (TEM) observations also revealed consistent results, where elements such as Ti, C, O, F, and Cl are evenly distributed on the nanosheet surfaces (Figure 1d). Additionally, atomic force microscopy (AFM) assessments determined the MXene nanosheets' thickness to be ≈1.3 nm (Figure 1e). This is consistent with prior reports, albeit slightly above the theoretical thickness of ≈1.0 nm.^[^ ^29^ ^]^ Such discrepancies can be attributed to factors such as the testing substrate and the terminal functional groups of MXene, suggesting the nanosheets are predominantly monolayered. The successful synthesis of MXene was further confirmed by X‐ray diffraction (XRD) analyses (Figure S4, Supporting Information). In comparison to its precursor Ti_3_AlC_2_, the MXene exhibits a complete disappearance of the 39° (104) diffraction peak, a broadening of the (002) diffraction peak, and a shift toward lower angles. The corresponding cross‐section reveals a typically dense, stacked, layered morphology characteristic of 2D materials (Figure 1f), aligning with previous reports.^[^ ^12^ ^]^

**Figure 2:**
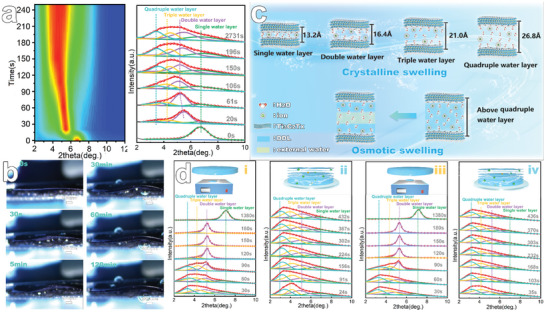
Mechanism of reversible dynamic dissociation of MXene film. a) In situ XRD patterns of MXene films immersed in water and fitting analysis of their diffraction peaks. b) Cross‐sectional alterations of the MXene film during water infiltration. c) Mechanism of the constrained dissociation of MXene film. d) XRD patterns of MXene film during cyclic in situ infiltration and quasi‐in situ drying.

To elucidate the reversible constrained dissociation and reformation mechanisms of MXene films, we utilized in situ XRD techniques, complemented by cross‐sectional optical morphological analyses. Upon immersion of the MXene films in water, a rapid influx of water molecules into the nanosheet interlayers was observed. With extended infiltration time, a discernible shift of the (002) diffraction peak of the MXene film to a lower angle was evident, indicative of increased interlayer spacing. Interestingly, this shift demonstrated a swift kinetic response (Figure S5, Supporting Information). Specifically, the (002) diffraction peak of the MXene film diminished from an initial 6.7° to 5.4° within just 20 s of infiltration, stabilizing ≈196 s. Through fitting analysis of the diffraction peaks, it was found that the increase in interlayer spacing is not continuous (**Figure** **2**a). In contrast, it exhibits characteristics similar to montmorillonite, with the interlayer spacing increasing non‐continuously as the number of water molecules augments.^[^ ^23^, ^24^ ^]^ Specifically, the associated interlayers comprised a single water layer (d = 1.32 nm), a double water layer (d = 1.64 nm), a triple water layer (d = 2.1 nm), and a quadruple water layer (d = 2.68 nm).^[^ ^30^, ^31^ ^]^ Figure 2b, alongside Video S1 (Supporting Information), captures the cross‐sectional changes of the MXene film during water infiltration. During this process, the film thickness initially expands rapidly, then gradually levels off, eventually exhibiting a typical logarithmic growth trend (Figure S6, Supporting Information). Based on these observations, the wetting disassociation process of the MXene film indeed appears similar to the swelling behavior of montmorillonite, mainly involving two stages: crystalline swelling and osmotic swelling.^[^ ^32^, ^33^ ^]^ This mechanism is more intuitively illustrated in Figure 2c. It is noteworthy that during the etching process of Ti_3_AlC_2_ to prepare Ti_3_C_2_T*_x_* MXene, hydrated Li^+^, driven by electrostatic forces on the nanosheet surface, spontaneously intercalate between the nanosheets, facilitating interlayer insertion and aiding subsequent rapid delamination. Importantly, the presence of these ions renders the interlayer spacing of the nanosheets to exhibit non‐fixed characteristics. For instance, we observed that the interlayer spacing differences within stacked MXene film ranged from 1.1 to 1.6 nm. Furthermore, propelled by the high hydration energy of interlayer Li^+^ (520 Kj mol^−1^) and the low diffusive resistance of the Ti_3_C_2_T*_x_* surface groups to water molecules, water can readily penetrate between the nanosheets, leading to enhanced ion hydration.^[^ ^22^, ^34^, ^35^ ^]^ Driven by this wedging force, the distance between nanosheets continues to increase. As the interlayer spacing increases, more water molecules are driven into these spaces, forming stable hydrogen bonds with the surface groups of the nanosheets, supplanting the original weak van der Waals forces between them. This leads to the formation of ordered lamellar structures of water molecules within the confined spaces of the 2D nanosheets, causing the water molecules in the MXene film to transition from a monolayer structure to a crystalline swelling process featuring multiple coexisting layers.^[^ ^24^ ^]^ However, when the number of water layers between the nanosheets exceeds four, the interlayer spacing becomes overly large, disrupting the electrostatic adsorption balance between the hydrated ions and the nanosheets on both sides. This phenomenon prompts the ions to form a diffusion double layer with a concentration gradient on both sides of the nanosheets, ultimately transitioning from crystalline swelling to osmotic swelling.^[^ ^36^ ^]^ As the nanosheet spacing widens and the interlayer water molecule count increases, the bilayer's repulsive force diminishes, decelerating the film's thickness growth rate.^[^ ^28^ ^]^

In the in situ XRD patterns, it is noteworthy that diffraction peaks for water molecules spanning more than four layers were absent. This absence can be attributed to reduced expansion pressure between the nanosheets, which prevents water molecules beyond the fourth layer from generating sufficient intra‐layer pressure for orderly alignment, resulting in the disappearance of the diffraction peaks.^[^ ^27^, ^37^ ^]^ Optical microscopy observations supported this hypothesis. After 2 h of infiltration, the film's cross‐sectional average thickness reached ≈99 µm (Figure S6, Supporting Information), significantly exceeding the calculated thickness of 10.6 µm based on the stable XRD (002) diffraction peak interlayer distance (2731 s). This indicates that during infiltration, the water molecule count between certain nanosheets exceeded four layers, suggesting a higher degree of dissociation. Furthermore, the selective infiltration of water molecules into the interlayer spaces of MXene suggests that, during the wetting process, certain regions of monolayer, bi‐layer, tri‐layer, and tetra‐layer hydration remain inaccessible to some water molecules. This results in only minor shifts in the XRD diffraction peaks throughout the wetting process, with these peaks remaining detectable at equilibrium.^[^ ^22^ ^]^ The sporadically spaced layers with lower interlayer hydration are stabilized by electrostatic interactions between the hydrated ions and nanosheet surfaces, ensuring that the MXene film does not spontaneously disintegrate but exhibits a state of restricted dissociation.^[^ ^12^ ^]^ This clarifies the pronounced warping and deformation observed in the film cross‐section in the early stages of wetting, which gradually diminishes as the wetting time progresses (Figure 2b; Video S1, Supporting Information). In fact, during the transition from lower to higher numbers of interlayer water molecules, variations in the rate at which the inter‐nanosheet spacing expansion across different regions of the film induces internal stresses, leading to deformation. As water molecules continue to infiltrate and their distribution becomes more uniform, these stresses are alleviated, permitting the film to regain its smoothness.^[^ ^38^ ^]^ This observation is corroborated by the enhanced smoothness observed on the restructured equilibrium film surface, as evidenced in Figure S7 (Supporting Information). Additionally, the dynamic dissociation and reversible transformation of infiltrated stacked MXene films were confirmed through cyclic in situ infiltration and quasi‐in situ drying (Figure 2d). It is evident that upon drying, the wetted MXene film, under the combined effects of surface tension from evaporating water, interlayer electrostatic, and van der Waals forces, transitions from a high integer layer of water molecules back to a low integer layer, ultimately reverting to a monolayer of water molecules,^[^ ^39^, ^40^ ^]^ and subsequently re‐establishing the 2D nanosheet stacking structure (Figure S8, Supporting Information). This mechanism resembles the behavior of slime molds coalescing in dry settings and dividing in moist conditions. This suggests that through water infiltration, MXene's densely packed macro‐assemblies can be endowed with a dynamic dissociation transition akin to cellular division in living organisms, facilitating the secondary reconfiguration of their original structures. This unique reversible infiltration‐dissociation characteristic of MXene opens avenues for tailored designs in layered spaces, distinctive micro‐particle characterizations within confined 2D spaces, and insights into confined chemical reactions.^[^ ^41^ ^]^

**Figure 3:**
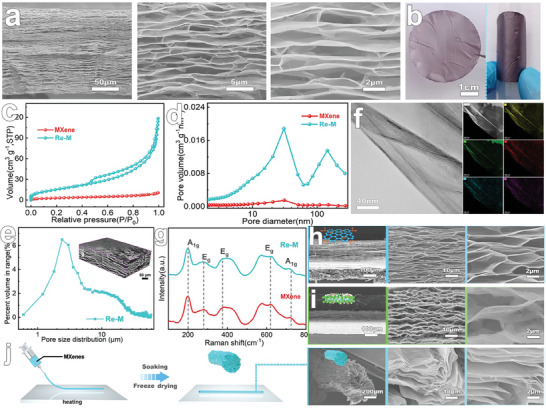
Structural characteristics of reconstructed Porous MXene films. a) Cross‐section morphology of Re─M. b) Photograph of Re─M and its bending. c) N_2_ adsorption/desorption isotherms of MXene film and Re─M. d) Pore size distribution of MXene film and Re─M. e) 3D‐reconstructed void microstructure derived from nano‐CT for Re─M. (f) TEM image and element distribution of Re─M. g) Raman spectra of MXene film and Re─M. h) Cross‐section morphology of Re─GO. i) Cross‐section morphology of Re─V. j) Schematic diagram of preparing M‐fiber through thermal drying and Cross‐section morphology of Re─M‐fiber.

By capitalizing on the wetting dynamic dissociation characteristics of MXene films and integrating rapid freezing techniques, we have successfully achieved a secondary reconstruction of internal porous structures within densely packed MXene films. Initially, water molecules penetrate specific interlayer nanosheet spacings under the influence of intercalation ion hydration energies, resulting in the formation of low integer water molecule layers in confined spaces.^[^ ^34^, ^35^ ^]^ As the interlayer spacing broadens, an imbalance in the electrostatic interactions between the hydrated ions and both sides of the nanosheets emerges. This induces selective adsorption on either side of the nanosheet, establishing a diffuse double layer. Such adsorption further widens the interlayer spacing, leading to the expansion of water molecules within the layers into disordered states, thereby effectuating the restricted dissociation of the MXene film.^[^ ^36^, ^37^ ^]^ Subsequently, during the rapid freezing process, ice crystals, constrained by the nanosheets, grow longitudinally, culminating in the formation of a continuous 3D porous film with a radial distribution resembling ‘cell walls’. This reformed porous MXene film (Re─M) distinctly diverges from its original counterpart in XRD patterns, displaying both the usual characteristic peaks and a notable (110) peak at 61°, indicative of a 3D porous structure (Figure S9, Supporting Information).^[^ ^42^ ^]^ Furthermore, the cross‐section of the film reveals an abundance of “cell wall”‐like structures. These wedge‐shaped formations, organized radially within the film, culminating in a macroporous arrangement that significantly amplifies the film's total thickness to ≈150 µm (**Figure** **3**a)—a nineteen‐fold increase compared to the original MXene film's thickness. Notably, these radially arranged pores form a stable and continuous 3D network structure by sharing the nanosheet pore walls, thereby endowing Re─M with remarkable flexibility.^[^ ^43^ ^]^ As shown in Figure 3b, Re─M, with a diameter of 40 mm, can be flexed and wrapped around the exterior of a centrifuge tube with a diameter of 16 mm. Moreover, this porous architecture significantly enhances the specific surface area of the MXene film, with Re─M attaining a specific surface area of 57 m^2^ g^−1^, representing a fivefold increase compared to the original MXene film (Figure 2c). Furthermore, we conducted a comprehensive analysis of these pore structures utilizing nitrogen isothermal adsorption‐desorption assays and Nano‐CT. The results indicate that Re─M contains mainly mesopores of ≈30 nm and also features a significant number of continuously distributed macropores with radial distribution, ranging from several hundred nanometers to a few micrometers or even tens of micrometers (Figure 3d,e). The genesis of these porous structures can be traced back to the rapid condensation nucleation and growth of numerous water molecules among the nanosheets, following the infiltration and dissociation of the densely stacked film during the rapid cooling process with liquid nitrogen.^[^ ^44^ ^]^ Owing to the accelerated cooling rate and nanosheet confinement, ice crystals preferentially grow in the nanosheets' radial direction, acting as templates that displace the nanosheets while interlinking through hydrogen bonding and van der Waals forces, culminating in the formation of multi‐scale pores aligned along the film's radial direction.^[^ ^42^ ^]^

It is noteworthy that the elemental composition of Re─M encompasses Ti, C, O, F, and Cl, which maintain a consistent distribution within the assembled and stacked nanosheets. Moreover, the elemental composition and ratio closely align with those observed in the original MXene film (Figure 3f; Figure S10a, Supporting Information). Simultaneously, no obvious changes were observed in the binding energy states of the elements or in the Raman spectral analysis (Figure 3g; Figure S10b, Supporting Information). This suggests that the dissociation and reconstruction process predominantly alters the 3D morphology of the assembly unit nanosheets without affecting their chemical composition. These findings clearly demonstrate that through simple water infiltration, MXene can be endowed with cell division and transformation capabilities reminiscent of living organisms, enabling the secondary reconstruction of the internal microstructure of macroscopically assembled MXene films.^[^ ^25^ ^]^ It should be noted that the reconfigured porous films lost their dissociable ability, as confirmed by the retention of the 3D porous characteristic diffraction peaks and structure after immersion in water (Figures S11 and S12, Supporting Information). This phenomenon could be attributed to the strong interactions between the MXene lamellae, resulting from the excessive removal of interlayer water molecules during vacuum freeze‐drying.^[^ ^23^ ^]^ This process creates a tightly interlocked layering that prevents water molecule insertion—akin to our prior observations about MXene films' time‐sensitive weldability.^[^ ^39^ ^]^ Consequently, Re─M retains its rich porous structure even upon exposure to water, addressing challenges in MXene liquid‐phase reactions, catalysis, and the preservation of porous support structures and properties in aqueous electrolytes. Interestingly, this dissociative reconstruction strategy is not limited to MXene but can be extended to graphene oxide (GO) films, other MXene variants (V_2_CT*_x_*) films, and MXene fibers. For instance, the cross‐section of the reconfigured GO film (Re‐GO) also features wedge‐shaped macropores distributed along the radial direction of the film, attaining a thickness of up to 220 µm—≈13 times that of the pristine GO film (Figure 3h; Figure S13, Supporting Information). Furthermore, the stacked V_2_CT*_x_* films, prepared through HF etching followed by TMA^+^ intercalation stripping (Figure S14, Supporting Information),^[^ ^45^ ^]^ and the MXene fibers (M‐fiber) fabricated via thermal drying (Figure S15, Supporting Information),^[^ ^46^ ^]^ exhibited dynamic dissociation and porous structural reconfiguration upon water infiltration and rapid freezing (Figure 3i,j; Figures S16–S18, Supporting Information). The aforementioned results underscore that this reversible, restricted dissociation property is an inherent phenomenon in hydrophilic 2D materials. This phenomenon can be judiciously harnessed to modify and utilize hydrophilic 2D materials across multiple dimensions. For instance, the dynamic dissociative transformation of films and fiber‐like formations in 2D materials can be achieved through water infiltration. This facilitates the secondary reconstruction of the internal microstructure, holding transformative significance for advancing the transition of 2D materials from laboratory settings to industrial applications.^[^ ^3^ ^]^

**Figure 4:**
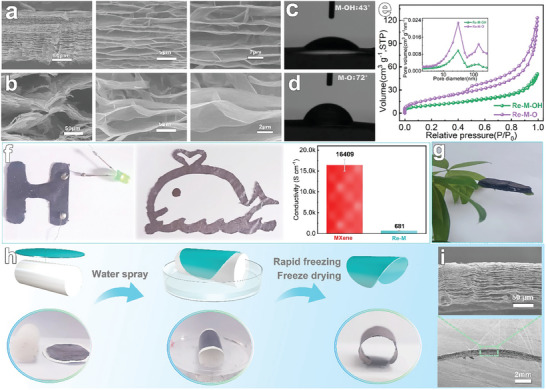
Strategic design of pore structures for reconstructed MXene films. a) Cross‐section morphology of Re─M─OH. b) Cross‐section morphology of Re─M─O. c) The wetting angle of M─OH. d) The wetting angle of M‐O. (e) N_2_ adsorption/desorption isotherms and pore size distribution of Re─M─OH and Re─M─O. f) Tetter H and whale patterns obtained by cropping and blade‐coating reconstruction and its conductivity. g) Photograph of MXene aerogel prepared by reconstruction of welding MXene film. h) Schematic illustration of the reconstructed porous MXenes films attached to different structural surfaces. i) Cross‐section morphology of reconstructed MXenes film with independently supported structures.

Given the significant impact of surface groups on the physicochemical properties of MXene, we specifically undertook alkalization and annealing processes to modulate the ‐F and oxygen‐containing groups at the terminals of MXene nanosheets, with the objective of modifying their surface ‐OH and ‐O groups.^[^ ^47^, ^48^ ^]^ The outcomes of these modifications were verified through XPS, Raman, and FTIR analyses (Figure S19, Supporting Information). XPS elemental analysis revealed that the F/Ti content ratio in M‐OH and M─O was significantly reduced compared to the MXene film, whereas the O/Ti ratio was substantially increased, indicating the successful removal of −F groups and incorporation of oxygen‐containing groups on the nanosheets' surface as a result of the alkalinization and annealing processes. An increased proportion of Ti─OH bonding peaks in M─OH, coupled with more pronounced ‐OH vibration peaks in Raman and infrared spectra, and a greater proportion of Ti‐O bonding peaks in M─O, with more intense −O vibration peaks, further corroborate the modification effects of alkalization and annealing on the −OH and −O groups on the MXene nanosheets' surface.^[^ ^49^, ^50^, ^51^, ^52^, ^53^ ^]^ This strategy further facilitated the exploration of the dissociation and reconstruction characteristics of the resulting functionalized MXene films. It shows that all of these functionalized MXene films possess the capability to dissociate and reconfigure, thus acquiring 3D porous structures (**Figure** **4**a,b; Figure S20, Supporting Information). Importantly, significant differences in thickness and pore morphology were observed among the modified MXene films, highlighting the impact of functionalization on their microstructural properties. The cross‐sectional distribution of the ‐OH‐modified reconstituted MXene film (Re─M─OH) closely mirrored that of the pristine MXene film, characterized by wedge‐shaped macropores distributed along the radial direction of the film (Figure S21, Supporting Information). In contrast, the ‐O‐modified reconstructed MXene film (Re─M─O) exhibited a significantly more porous internal structure and a markedly higher cross‐sectional thickness, achieving up to 35 times that of the original structure. Furthermore, Re─M─O demonstrated a significantly higher specific surface area, amounting to 68.2 m^2^ g^−1^, compared to both Re─M─OH, which exhibited 36.9 m^2^ g^−1^, and the pristine MXene film (Figure 4e). We speculate that the observed microstructural differences are closely correlated with variations in the Zeta potential and wettability characteristics of the nanosheet surface groups. It was observed that the Zeta potential of MXene stood at −64.5 mV, which decreased to −75.9mV following hydroxyl modification (M‐OH), whereas the Zeta potential of the oxygen‐containing group modified MXene (M─O) increased to −37.9mV (Figure S22, Supporting Information). This difference in potential corresponds well with the porous structures of the reconstituted steady‐state films (Re─M, Re─M─OH, and Re─M─O). Among them, the notably negative potential of M─OH enhances the electrostatic interaction between the nanosheets and the interlayer ions during the wetting dissociation process. This enhancement inhibits film dissociation. Additionally, its low wetting angle (43°, Figure 4c,d; Figure S23, Supporting Information) facilitates a more uniform distribution of interlayer water molecules, culminating in the reconstituted film exhibiting more uniform pores and minimal thickness. This hypothesis received further support from the observed enhanced structural stability of Re─M─OH under an identical amplitude of shaking post‐infiltration, in comparison to its counterparts (Figure S24 and Video S2, Supporting Information). Conversely, annealing led to an increase in the surface potential of M─O to −37.9mV, diminishing the electrostatic interaction with intercalated ions during the wetting process. This alteration enhances the dissociation capability of the stacked films and may lower the critical water layer threshold necessary for the transition from crystalline to osmotic swelling. Furthermore, its elevated wetting angle (72°) promotes aggregation of interlayer water molecules, culminating in the reconstituted Re─M─O film exhibiting the most significant thickness and larger pores.^[^ ^21^, ^54^ ^]^ Consequently, the strategic design of pore structures within reconfigured MXene films can be adeptly realized through the relatively well‐established methodologies of nanosheet surface group modulation and dynamic dissociation strategies. Certainly, porous reconfiguration of the complex patterns of the blade coated MXene films is also easily achieved by the infiltration dissociation combined with the cropping process. As shown in Figure 4f, the letter H and whale patterns obtained by cropping and blade‐coating reconstruction, respectively, and the continuous conductive network formed by interconnected nanosheets within the film enables the conductivity of the expanded film to still reach 681 S cm^−1^, which is the highest value known for conductive porous 2D material films.^[^ ^55^, ^56^, ^57^ ^]^

Thick MXene films are critically needed in applications such as desalination and thermal insulation. However, the prevailing methods for preparing these thick films, primarily through filtration and self‐evaporation, are time‐consuming and elevate the risk of oxidation.^[^ ^5^, ^7^ ^]^ To address these challenges, we have successfully expanded the thickness of MXene films to the millimeter scale and formed independently supported aerogels of similar composition (Figure 4g; Figure S25, Supporting Information). This was achieved by dissociating and reconstructing the stacked films longitudinally, leveraging our previously reported water‐assisted welding technique.^[^ ^41^ ^]^ It's worth noting that conventional porous MXene design methods largely employ a ‘bottom‐up’ strategy, often resulting in a single structure of the produced porous MXene films. Achieving controlled construction of porous MXene films for macroscopic complex assemblies has emerged as a significant scientific hurdle for both academia and industry.^[^ ^58^ ^]^ Interestingly, this challenge can now be addressed by leveraging the reversible constrained dissociation and reconfiguration properties inherent to MXene films. By attaching MXene films to uniquely shaped surfaces prior to dynamic dissociation, we've been able to create 3D porous films that inherently adapt to the underlying substrate structure (Figure 4h; Figure S26, Supporting Information). Figure 4i illustrates a cross‐section of an MXene cylinder adhered to a 16 mm diameter polyethylene cylinder, showcasing wedge‐shaped holes curved along the cylinder. This is mainly due to the excellent flexibility of the nanosheets and the fact that the wetted film adheres to the substrate surface by surface tension to dissociate and release stress in situ, and the porous pore walls are rigidly supported by rapid freezing and drying, resulting in independently supported heterogeneous porous MXene films.^[^ ^43^, ^44^ ^]^ Such uniquely supported shaped films are anticipated to broaden the applicability of MXene porous structures in complex scenarios. Utilizing the dynamic dissociation and reconfiguration techniques of MXene films, along with the nanosheet surface matrix design, welding, and attachment techniques, we have successfully achieved precise control over both the microscopic pore structure and macroscopic morphology of reconfigured MXene films. This advancement significantly bolsters the potential for wide‐ranging applications of MXene in seawater desalination, electrocatalysis, and electrochemical energy storage domains.^[^ ^12^, ^13^, ^14^, ^15^, ^16^ ^]^

**Figure 5:**
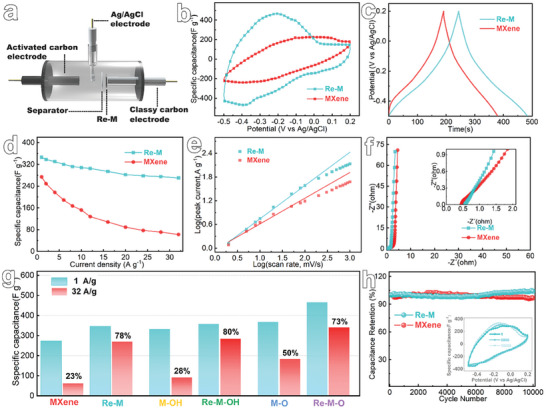
Electrochemical performance of reconstructed MXene films. a) Schematic illustration of three‐electrode system. b) CV curves of MXene film and Re─M at 50 mV s^−1^. c) GCD curves of MXene film and Re─M at 1 A g^−1^. d) Specific capacitance of MXene film and Re─M at different current density. e) Plots of peak current response of MXene film and Re─M measured against scan rate. f) Nyquist plots of MXene film and Re─M. g) Specific capacitance of reconstructed porous MXene films and its competitive restacking film. h) Capacitance retention test of MXene film and Re─M.

Since its introduction in 2011, MXene has shown significant promise in various fields, with its application in electrochemical energy storage standing as a crucial step toward commercialization.^[^ ^8^, ^9^ ^]^ Presently, engineering 2D MXene nanosheets into porous structures has been identified as an effective strategy to mitigate its notable aggregation issue and improve ion transport kinetics.^[^ ^59^ ^]^ To evaluate the electrochemical properties of our reengineered porous MXene film, we conducted specialized tests on their electrochemical capacitance within a three‐electrode system (**Figure** **5**a). Both the MXene film and Re─M displayed characteristic CV and constant‐current charge–discharge (GCD) curves—depicting distorted rectangles and triangles, respectively. The distortion is induced by the pseudocapacitance resulting from the coupling of hydrogen ions with oxygen‐containing groups on the nanosheet's surface, which leads to the alteration of the valence state of titanium (Ti) elements (Figure S27, Supporting Information).^[^ ^11^, ^16^ ^]^ The 3D porous architecture of Re─M markedly improves the accessibility of electrolyte ions to active surface sites. This enhancement is manifested through more distinct redox peaks, a broadened integration area (Figure 5b), and prolonged charge/discharge durations (Figure 5c). At a scan rate of 2 mV s^−1^, Re─M exhibited a specific capacitance of 345 F g^−1^, significantly surpassing the capacitance of the MXene film (279 F g^−1^, Figure S28, Supporting Information). Similarly, at a current density of 1 A g^−1^, the capacitance of Re─M stands at 346 F g^−1^, significantly exceeding that of the MXene film at 274 F g^−1^ (Figure 5d). These metrics notably exceed most previously documented values for both optimized and native MXene structures (Table S1, Supporting Information). Moreover, the rate performance of Re─M is notably enhanced, owing to its optimized pore structure facilitating a more rapid ion diffusion pathway.^[^ ^39^, ^42^ ^]^ The capacitance retention reaches up to 78% (270 F g^−1^) at an increased current density of 32 A g^−1^, much higher than the 23% (62 F g^−1^) of the MXene film. A deeper dive into the kinetics of the film's charge storage process was undertaken using the relationship between scan rate (v) and peak current (Equation S8, Supporting Information), where a b‐value of 1 signifies non‐diffusion‐controlled double‐layer capacitance and surface pseudocapacitance mechanisms, while a b‐value of 0.5 points to the diffusion‐controlled paradigm.^[^ ^44^ ^]^ The derived b values were 0.84 for Re─M and 0.65 for MXene film, respectively (Figure 5e). These values suggest that the porous architecture of Re─M predisposes it more toward the non‐diffusion‐controlled surface capacitance energy storage mechanism, when compared to the MXene films. Figure 5f presents the AC impedance spectra of both Re─M and MXene film. Despite the reduction in nanosheet contact due to the porous structure, Re─M sustains a continuous conductive network, courtesy of the extrusion process utilizing the ice crystal template, thereby exhibiting a remarkably low internal resistance of 0.52 Ω.^[^ ^43^ ^]^ Due to the open multi‐scale pores, larger exposed active sites, and continuous conductive network inside Re─M, this ensures efficient carrier transfer during charging and discharging, resulting in an extremely low charge transfer resistance. The steeper slope of Re─M in the low‐frequency region, in contrast to the MXene film, further signifies that its porous architecture substantially mitigates ion diffusion resistance and enhances ion transport kinetics, ultimately translating to superior electrochemical energy storage performance. Furthermore, the electrochemical performances of the dissociated and reconstituted 3D porous films have experienced noteworthy enhancements through the optimization of surface oxygenated groups and the amplification of surface active sites (Figures S29 and S30, Supporting Information).^[^ ^48^ ^]^ Specifically, at a current density of 1 A g^−1^, Re─M─OH and Re─M─O displayed specific capacities of 358 and 465 F g^−1^, respectively, marking increases of ≈8% and 26% compared to their stacked counterparts. Additionally, upon increasing the current density to 32 A g^−1^, the capacitance retention for Re─M─OH and Re─M─O surged to 80% and 73%, respectively, which are 2.9 and 1.5 times higher than that of the stacked structure (Figure 5g), showcasing outstanding rate performance. Cycling stability is a crucial indicator for assessing the suitability of supercapacitor electrode materials for practical applications. Post 10 000 charge–discharge cycles, Re─M not only maintained its cycling stability but also manifested a slight increase in specific capacity (Figure 5h). This enhancement can likely be attributed to the expanded layer spacing resulting from hydrogen ion insertion during charging, consequently enhancing the contribution from insertion pseudocapacitance.^[^ ^60^ ^]^

To evaluate the potential of reconfigured porous‐structured films for practical applications, we assembled Re─M‐based symmetric supercapacitors and tested their energy density, power density, and cycle life. Comparative analyses reveal that Re─M exhibits a larger integration area and extended discharge times at equivalent scan rates and current densities than MXene film, demonstrating its superior capacitance performance and rate characteristics (Figure S31 and S32, Supporting Information).^[^ ^59^ ^]^ At a power density of 176.5 µW cm^−2^ (30.3 W kg^−1^), the Re─M symmetric device achieves an areal energy density of 24.5 µWh cm^−2^ (4.2 Wh kg^−1^). Owing to the enhanced ion transport kinetics afforded by its reconfigured porous structure, even when the power density is increased to 26630.4 µW cm^−2^ (4593.7 W kg^−1^), the Re─M device sustains an areal energy density of 7.4 µWh cm^−2^ (1.3 Wh kg^−1^), which is significantly better than that of the MXene film and the majority of the previously reported symmetric devices made of MXene and other 2D materials devices (Figure S33a, Supporting Information). Importantly, there is almost no loss of specific capacity after 6000 charge/discharge cycles (Figure S33b, Supporting Information), which shows that dissociated and reconfigured multiscale porous MXene has great potential for practical applications in electrochemical energy storage.

**Figure 6:**
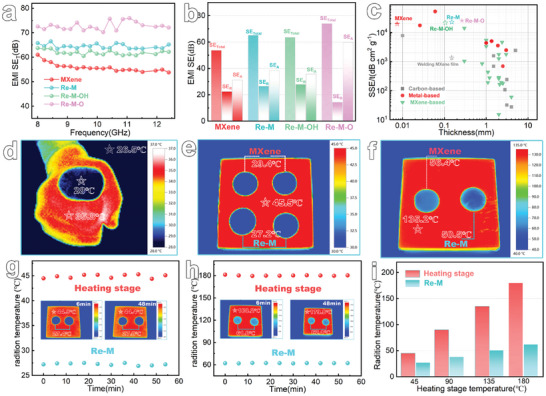
Electromagnetic interference shielding and infrared stealth performances of reconstructed MXene films. a) EMI shielding effectiveness of MXene film, Re─M, Re─M─OH and Re─M─O. b) Total EMI shielding effectiveness and corresponding absorption, a reflection of MXene film, Re─M, Re─M─OH and Re─M─O. c) Comparison of the EIM shielding effectiveness for reconstructed porous MXene film with previously reported carbon‐based, metal‐based, MXene‐based. d) Infrared image of a Re─M placed on the fist. Infrared images of Re─M placed on a heating stage at e) 45°C and f) 135°C. (g) and (h) Infrared stealth performance of Re─M continuously placed on a heating stage at (g) 45°C and (h) 180°C. (i) Radiation temperatures of Re─M on a heating stage at different temperatures.

Amid the rapid advancement of global aerospace technology, there has been a marked increase in the demand for lightweight porous electromagnetic interference (EMI) shielding materials. To date, MXene films have been recognized as leading synthetic materials for EMI shielding performance.^[^ ^14^ ^]^ However, the dense stacking of MXene nanosheets within conventional MXene films results in a high density that does not meet the lightweight material requirements critical for aerospace applications.^[^ ^61^ ^]^ Presently, Re─M, crafted through dissociation and reconstruction techniques, is poised to propel the evolution of MXene materials within the EMI shielding domain further. The EMI shielding effectiveness (EMI SE) of the samples across the X‐band (8.2–12.4 GHz) was meticulously evaluated using a vector network analyzer. As shown in **Figure** **6**a, all samples exhibit slight frequency dependence regarding their EMI SE across the X‐band. In particular, the average EMI SE of Re─M reaches an elevated level of 65 dB, capable of blocking nearly 99.9999% of incident radiation, a significant improvement over the MXene film (53 dB). Notably, the average EMI SE of Re─M─O reaches 73 dB, due to its more developed internal porous structure providing more opportunities for multiple reflections and interface polarization of electromagnetic waves, resulting in more effective attenuation.^[^ ^62^ ^]^ Additionally, the enhanced conductivity due to annealing (Figure S34, Supporting Information) and dipole polarization losses caused by surface oxygen‐containing group modifications further increased electromagnetic wave absorption.^[^ ^29^, ^63^ ^]^ Moreover, the EMI SE of Re─M─OH is weaker than that of Re─M─O and Re─M, which is closely related to the difference in the arrangement of its internal porous structure. Figure 6b presents the correlation between microwave absorption (SE_A_), reflection (SE_R_), and the total EMI shielding effectiveness (SE_Total_) for MXene, Re─M, Re─M─OH, and Re─M─O. For Re─M and MXene film, although their SE_R_ values are comparable, Re─M's SE_A_ performance is superior to that of MXene film. The enhanced EMI shielding effectiveness of Re─M can be primarily attributed to the enhanced electromagnetic wave scattering at the interfaces of the internally reconstituted 3D porous pore walls, thereby extending the path of electromagnetic wave transmission.^[^ ^15^, ^64^ ^]^ In addition, for Re─M─O, there is a significant enhancement in its SE_A_, indicating enhanced electromagnetic wave absorption and attenuation capacity as the waves entered its developed porous structure. Absolute shielding effectiveness (SSE/*t*), integrating the material's density, thickness, and EMI SE, emerges as a crucial parameter for evaluating lightweight porous EMI shielding materials. As shown in Figure 6c, the SSE/*t* value of Re─M achieves 22,800 dB cm^2^ g^−1^, surpassing that of the MXene film (18,417 dB cm^2^ g^−1^) by 1.2 times and the water‐assisted welded MXene film with a thickness of ≈150 µm (1336 dB cm^2^ g^−1^, Figure S35, Supporting Information) by 17 times. The relationship between SSE/*t* and the sample thickness highlights Re─M's status as one of the best known synthetic materials for EMI shielding performance.^[^ ^14^, ^49^ ^]^ Additionally, the SSE/*t* values of Re─M─O and Re─M─OH were 25,298 and 21,510 dB cm^2^ g^−1^, respectively, demonstrating their potential application in the field of lightweight EMI shielding. Currently, it has been clearly established that MXene possesses an extremely low infrared (IR) emissivity, making it of great value in the field of advanced IR camouflage applications.^[^ ^65^ ^]^ To investigate the IR camouflage capabilities of samples, we observed the behavior of the samples on a heat source using an IR thermal imager. As shown in Figure 6d, the radiant temperature of Re─M, when placed on the fist, is markedly lower than that of the fist itself, aligning closely with the ambient temperature. Notably, the radiant temperature of Re─M consistently stays below that of the MXene film, both at milder temperatures (Figure 6e) and at elevated temperatures (Figure 6f). This suggests that Re─M possesses a superior thermal camouflage capability compared to MXene film, a feature primarily attributable to its rich, continuous porous structure, which contributes to enhanced thermal insulation capability.^[^ ^57^ ^]^ To delve deeper into the IR stealth performance of Re─M, it was subjected to continuous heating for 60 min at temperatures of 45°C (Figure 6g), 90°C (Figure S36a, Supporting Information), 135°C (Figure S36b, Supporting Information), and 180°C (Figure 6h). It is apparent that Re─M maintains its IR stealth capability across different temperatures irrespective of the heating duration and remarkably reduces the radiation temperature of the heating table from 180 to 60°C (Figure 6i). Additionally, Re─M─OH and Re─M─O also possess the ability to maintain infrared stealth over a long period, similar to Re─M. Notably, similar to EMI shielding, the IR stealth capability of Re─M─O is slightly improved compared to Re─M. For instance, at 135°C, the surface temperature of Re─M─O was only 47.3°C, lower than Re─M's 50.7°C (Figure S37, Supporting Information), primarily due to its larger porous structure providing certain insulation effects.^[^ ^66^ ^]^ The IR stealth capability of Re─M─OH is slightly inferior to Re─M and Re─M─O (Figure S38, Supporting Information), but still better than MXene film. This performance highlights the outstanding application potential of these porous MXene films in specialized military environments. Therefore, the reversible constrained dissociation and reconfiguration attributes of MXene films render MXene materials promising candidates for pivotal roles across a myriad of domains.

## 3 Conclusion

In summary, we have revealed the unique reversible transformation capability inherent in hydrophilic 2D materials, including MXenes and graphene oxide (GO). These materials exhibit a constrained dissociation characterized by crystalline swelling and osmotic swelling when infiltrated by water molecules, followed by a recovery of their stacked structures driven by surface tension and van der Waals forces during the drying process. This phenomenon not only emulates the fission and aggregation behaviors of living organisms but also holds significant importance for a deeper understanding of the complex interactions between 2D materials and water molecules. This insight opens new avenues for the development of novel interlayer confined chemical reactions and the optimization of ion transport in aqueous conditions. Furthermore, utilizing the principles of constrained dissociation and rapid water molecule freezing, we have ingeniously engineered an inverted 3D reassembly of MXenes' porous structures from the top down for the first time, yielding unconventional porous films and aerogels characterized by intricate patterns, flexibility, and standalone support. The simplicity, effectiveness, and broad applicability of this strategy underscore its vast potential in broadening the applications of 2D materials like MXenes in domains such as electromagnetic shielding, energy storage, and seawater desalination. By achieving precise structural control and functional integration, this work paves new pathways for the macroscopic assembly and application development of 2D materials.

## 4 Experimental Section

### Materials

The commercial Ti_3_AlC_2_ and V_2_AlC powders were sourced from Jilin 11 Technology Co., Ltd. Graphene oxide (GO) was procured from Suzhou Tanfeng Technology Co., Ltd. Concentrated hydrochloric acid (HCl, 36–38%) and sulfuric acid (H_2_SO_4_) were acquired from Guangdong Guangshi Reagent Technology Co., Ltd. Hydrofluoric acid (HF) solution (concentration >40%), tetrabutylammonium hydroxide (10% concentration), and other chemicals of analytical grade were commercially available from Aladdin Reagent, and were used as delivered, without any further purification.

### Synthesis of Monolayer MXene (Ti_3_C_2_T_x_) Dispersion

MXene was achieved through an improved minimally intensive layer delamination approach. Initially, the process commenced with the dissolution of 4 g of LiF into 80 mL of concentrated hydrochloric acid, under conditions of vigorous stirring. Subsequently, 4 g of Ti_3_AlC_2_ powder was gradually introduced into the mixture. The reaction assembly was then maintained at a constant temperature of 45°C, with persistent, over a duration of 48 h. Upon completion of this interval, the mixture was allowed to cool to ambient temperature and was left undisturbed for an additional 12 h. The transparent supernatant was delicately decanted, leaving behind the precipitate which was then subjected to multiple cycles of centrifugal washing with deionized water, until a neutral pH level was attained. This procedure resulted in a multilayered Ti_3_C_2_T*_x_* slurry. This slurry underwent further processing, being mixed with 400 mL of deionized water and subjected to ultrasonication in an ice bath for 1 h. Following ultrasonication, the mixture was centrifuged at 3600 rpm for 5 min, a step repeated thrice to ensure the complete separation of the monolayer nanosheets. The resultant monolayer Ti_3_C_2_T*_x_* nanosheet suspension was then collected. To determine the concentration of the nanosheet dispersion, a 5 mL sample was extracted and subjected to vacuum‐assisted filtration through a cellulose filter membrane. The filtered material was then dried at 60°C for 24 h and weighed, allowing for the determination of the suspension concentration.

### Dissociation and Reconstruction of MXene Film

The MXene film was fabricated through a filtration process, employing a dispersion of Ti_3_C_2_T*_x_* with a solid content of 40 mg, passed through a cellulose filter membrane of 2.1 cm radius. Post‐filtration, the resultant film was seamlessly detached from the filter paper after undergoing a drying process—initially at ambient temperature for 4 h, followed by a 24 h period at 60°C, culminating in the formation of the MXene film. For the dissociation and subsequent reconstruction of the MXene film, the procedure entailed fully immersing the fabricated MXene film in deionized water for 2 h, thereafter carefully removing the surplus water. Following this, the dissociated MXene film was immersed in liquid nitrogen for several minutes and then freeze‐dried, resulting in reconstituted porous MXene film, denoted as Re─M.

### Dissociation and Reconstruction of Surface Group‐Modified MXene Film

The MXene film was submerged in a 4 m KOH solution, with Ar gas employed to expel dissolved oxygen, and maintained in an Ar atmosphere for 1 h. This step was followed by a thorough rinsing of the film with deionized water until a neutral pH was attained. The film was then air‐dried for 4 h and subsequently oven‐dried at 60°C for 24 h, resulting in the formation of hydroxyl (‐OH) modified MXene film, denoted as M‐OH. Additionally, the oxygen‐containing group‐modified MXene film, designated as M─O, was prepared by annealing the MXene film at 400 °C under a N_2_ atmosphere. Referring to the dissociation and reconstruction process of MXene film, both M─OH and M─O were subjected to infiltration, followed by rapid freeze‐drying, to obtain the corresponding reconstructed porous MXene films, named Re─M─OH and Re─M─O, respectively.

### Reconstruction of GO Fim, V_2_CT_x_ fim and Ti_3_C_2_T_x_ Fibers

Graphene oxide (GO) film was prepared with reference to the the preparation of MXene film. For V_2_CT*_x_* film, the process began with immersing 4 g of V_2_AlC into 60 mL of HF solution and stirring at ambient temperature for an extensive period of 72 h. After allowing the mixture to settle for 2 h and removing the supernatant, the remaining slurry was rinsed with deionized water to achieve a neutral pH. The slurry was then treated with 40 mL of an aqueous solution containing 3 mL of tetrabutylammonium hydroxide, stirred in an ice bath under an Ar atmosphere for 6 h. The resulting upper suspension was collected through vortex shaking for 3 min and subjected to centrifugation at 3600 rpm for 5 min, a process repeated four times to procure the purified V_2_CT*_x_* dispersion. V_2_CT*_x_* film was then prepared with reference to the preparation of MXene film. For Ti_3_C_2_T*_x_* fiber, a 50 mL of Ti_3_C_2_T*_x_* dispersion was mixed with 200 mg of NaCl, and thoroughly dissolved through vigorous shaking. Centrifugation at 10 000 rpm for 5 min facilitated the collection of the dense slurry at the bottom, which was further concentrated via filtration. The concentrated slurry was then extruded through a 27G syringe needle onto a heating table maintained at 60°C for 10 min and dried further at the same temperature for 24 h to yield Ti_3_C_2_T*_x_* fiber, hereafter referred to as M‐fiber. Referring to the dissociation and reconstruction process utilized for MXene films, the aforementioned GO film, V_2_CT*_x_* film, and M‐fiber were subjected to infiltration, dissociation, and rapid freeze‐drying to obtain the corresponding reconstituted materials, named Re‐GO, Re‐V, and Re─M‐fiber, respectively.

### Characterization Methods

The composition phases of the samples were determined using a Rigaku Ultima IV X‐ray diffractometer (XRD) equipped with Cu Kα radiation (λ = 0.154 nm), covering a scan range from 4° to 70°. The morphology was examined via a Hitachi SU‐8010 scanning electron microscope (SEM), and the microstructure was verified using a Tecnai G2 F30 transmission electron microscope (TEM). The chemical composition of the samples was identified with an ESCALAB 250Xi X‐ray photoelectron spectrometer (XPS) utilizing Al Kα radiation (hν = 1486.6 eV) at an acceleration voltage of 15 kV, with all samples being sputter‐coated with Ar ions for 60 s before examination. Raman spectroscopy analysis was performed using an inVia Reflex Raman spectroscope with a 532 nm laser. The surface area, according to the Brunauer–Emmett‐Teller (BET) method, was measured with a Micromeritics ASAP 2020 system. Nanoscale X‐ray computed tomography (nano‐CT) was performed using a ZEISS Xradia 800 Ultra with an 8 keV Cu‐Kα X‐ray source. The Zeta potential was measured by a Zeta potential analyzer (Horiba SZ‐100). Before testing, the stacked MXene film, M─OH, and M─O were dispersed in water for 10 min through ultrasonication to obtain a suspension. The termination group of samples was identified with a Nicolet Nexus 670 FTIR. In situ XRD analysis employed a Rigaku SmartLab X‐ray diffractometer with a Cu Kα radiation source (λ = 0.154 nm), operating at a scan rate of 30°min within a 2–12° range. For the testing procedure, two layers of cellulose filter paper, soaked in deionized water, were cut to fit within the glass molds and placed accordingly. A MXene film, sized to match the filter paper, was then layered on top, with deionized water added to just below the level of the MXene film. The test commenced swiftly, with additional deionized water supplied once during the test to counter natural evaporation. Upon completion, the glass molds housing the dissociated samples were removed, excess water was absorbed using qualitative filter paper, and the samples were placed on a heating table set at 60°C, with testing carried out at 30 s intervals. The in situ dissociation and quasi‐in situ drying were cycled as described to verify the reversibility of MXene film infiltration dissociation. In situ optical microscopy observation was performed using a WYT‐ET body microscope and a WY‐630 high‐definition digital camera, with the MXene film cross‐section rendered brittle via liquid nitrogen exposure. For the electromagnetic shielding test, an Agilent N5244A PNA network analyzer was utilized, operating within an X‐band frequency range of 8.5‐12.4 GHz, the EMI test samples in this work were measured using uniform sizes and weights, with the test films having diameter of ≈42 mm and a weight of ≈40 mg. The infrared thermal radiation temperature of the prepared samples was detected using a TESTO 865 infrared thermal imager.

### Electrochemical Measurements

Electrochemical evaluations were systematically carried out using a Swagelok‐type three‐electrode configuration within a 3 m H_2_SO_4_ aqueous electrolyte. The investigations employed the as‐synthesized samples, each with an approximate diameter of Φ3 mm, as the working electrode. A platinum foil electrode and a Ag/AgCl electrode were utilized as the counter and reference electrodes, respectively. Electrochemical analyses, including cyclic voltammetry (CV), galvanostatic charge–discharge (GCD), and electrochemical impedance spectroscopy (EIS), were conducted on a CHI 760E electrochemical workstation (Chenhua Instruments, Shanghai, China). For the CV test, the gravimetric capacitance (C_g_) of the electrode was determined using the equation:

(1) $$\mathrm{Cg}\;=\frac{\int \mathrm{Idv}}{\upsilon \times V\times m}\;$$

where *C_g_* (F g^−1^) is the gravimetric capacitance of the electrode, *I* (A) is the charge–discharge current, *υ* (V s^−1^) is the scan rate, *m* (g) is the mass of the working electrode. *V* (V) is the voltage window.

For the GCD test, the *C_g_* of an electrode can be calculated using the given equation:

(2) $$\mathrm{Cg}\;=\frac{I\Delta t}{\Delta V\times m}\;$$

where *C_g_* (F g^−1^) is the gravimetric capacitance of the electrode, *I* (A) is the charge–discharge current, Δ*t* (s) is the discharge time, ΔV (V) represents voltage drop on discharging (excluding the IR drop), *m* (g) is the mass of the working electrode.

For the construction of symmetric supercapacitors, two identically prepared samples with equal mass loading were utilized, with a nonwoven fabric separator (NKK‐MPF30AC‐100) placed between them. The gravimetric energy density (*E_w_*, Wh kg^−1^) and areal energy density (*E_a_*, Wh cm^−2^) of the symmetric supercapacitor are calculated based on the following equations:

(3) $$E_{w}=\int \mathrm{VI}\;dt/m$$

(4) $$P_{w}=E_{w}\;/▵t$$

(5) S=A/m

(6) $$E_{a}=E_{w}\;/S$$

(7) $$P_{a}=E_{w}\;/S$$

where *V* represents the potential windows, *m* is the total weight of electrode materials, *I* is the current density, m and and ▵t (s) is the discharge time, *A* is an area of electrode material.

To delve into the charge storage kinetics, the relationship between the peak current(*i_p_*) and the scan rate (*v*) is explored through the formula:

(8) $$i_{p}=\;av^{b}$$

where a and b are variables, with the b‐value determined from the slope of the plot of log ip versus log v.

## Conflict of Interest

The authors declare no conflict of interest.

## Supporting information

Supporting Information

Supplemental Video 1

Supplemental Video 2

## Data Availability

The data that support the findings of this study are available from the corresponding author upon reasonable request.
