# Supplementary material for: Reversible Constrained Dissociation and Reassembly of MXene Films
Source: Adv Sci (Weinh). 2024 Apr 6;11(23):2309171. doi: 10.1002/advs.202309171 (PMC11186054; doi:10.1002/advs.202309171)
Supplement: Supplementary file 1 — Supporting Information [file ADVS-11-2309171-s001.pdf]

ADVANCED SCIENCE
Open Access

Supporting Information

for *Adv. Sci.*, DOI 10.1002/advs.202309171

Reversible Constrained Dissociation and Reassembly of MXene Films

Xuefeng Zhang, Xudong Liu, Qingqiang Liu, Yufa Feng, Si Qiu, Ting Wang, Huayu Xu, Hao Li, Liang Yin, Hui Kang and Zhimin Fan**

Reversible constrained dissociation and reassembly of MXene films

Xuefeng Zhang,[1] Xudong Liu,[2] Qingqiang Liu,[1] Yufang Feng,[1] Si Qiu,[1] Ting Wang,[1] Huayu Xu,[1] Hao Li,[1,*] Liang Yin,[3] Hui Kang,[4] Zhimin Fan,[2,3,5*]

[1]School of chemistry and Materials Engineering, Guangdong Provincial Key Laboratory for Electronic Functional Materials and Devices, Huizhou University, Huizhou 516007, Guangdong, China

[2]School of Materials Science and Engineering, Harbin Institute of Technology, Harbin, 150001, China

[3]MIIT Key Laboratory of Critical Materials Technology for New Energy Conversion and Storage, School of Chemistry and Chemical Engineering, Harbin Institute of Technology, Harbin 150001, China

[4]Advanced Materials Thrust, The Hong Kong University of Science and Technology (Guangzhou), 510000, Guangzhou, China

[5]Lead contact

*Corresponding author

E-mail addresses: fanzm@hit.edu.cn (Z.F.), lihao180@126.com (H. L.)

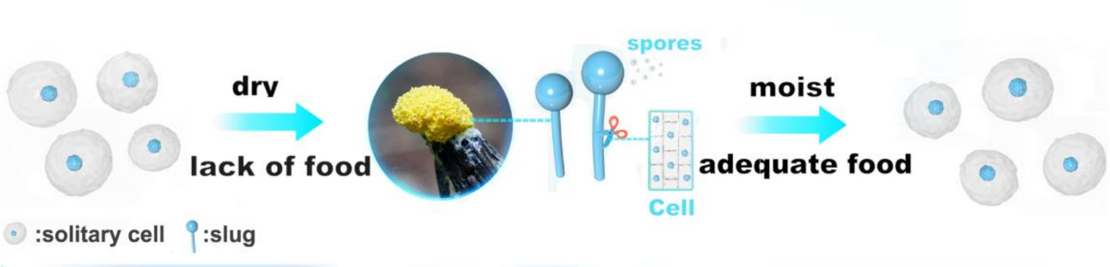

Figure S1. Schematic diagram of the chemotaxis of slime molds in arid conditions and their reproductive and divisional processes in humid environments.

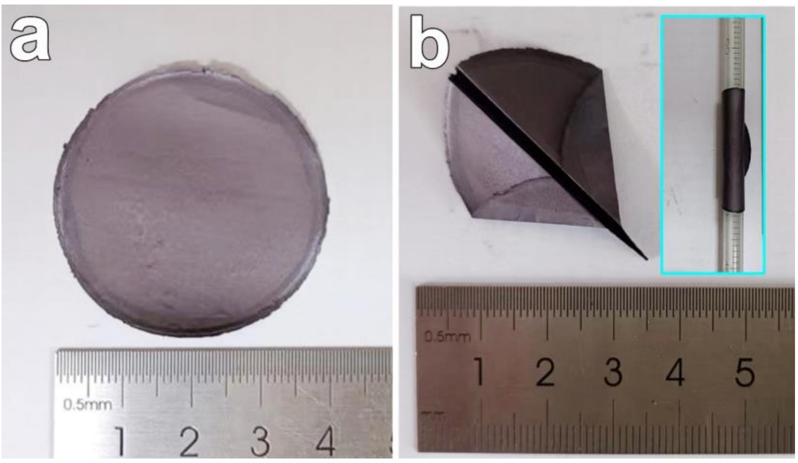

Figure S2. Optical morphologies of (a) the assembled MXene film by vacuum filtration and (b) their folded.

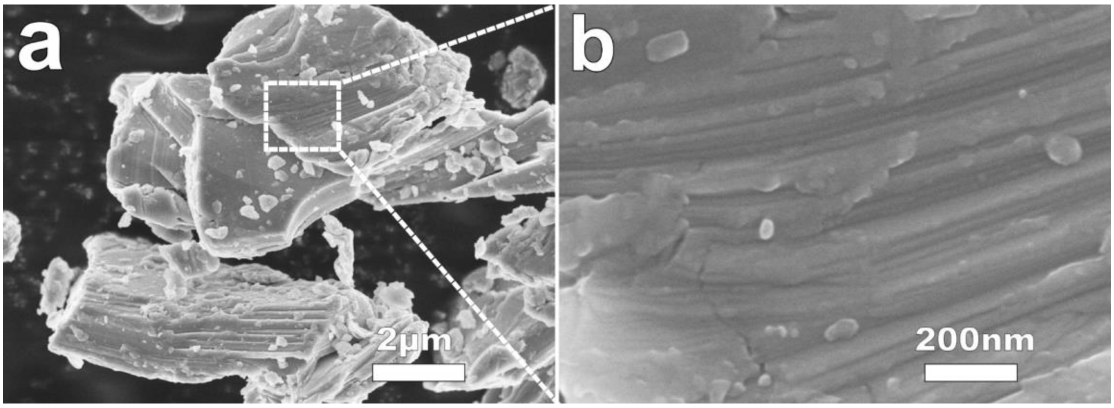

Figure S3. SEM images of Ti_3AlC_2 precursor, (a) low magnification; (b) high magnification.

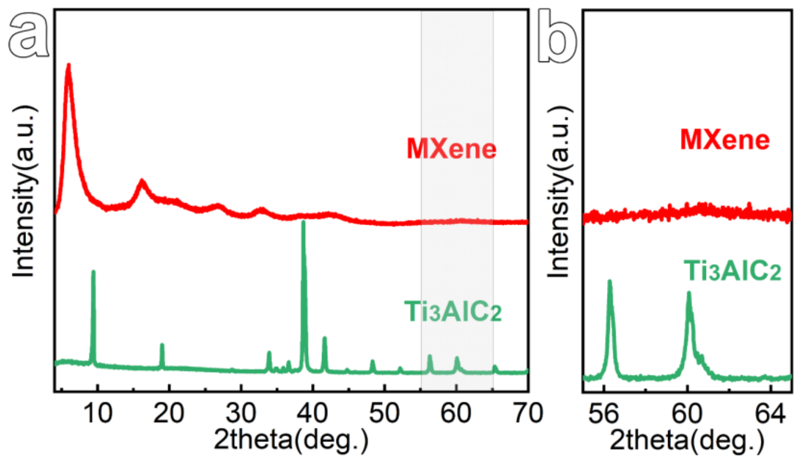

Figure S4. (a) XRD patterns of the Ti_3AlC_2 and MXene film and (b) their detailed XRD patterns around 55°-65°.

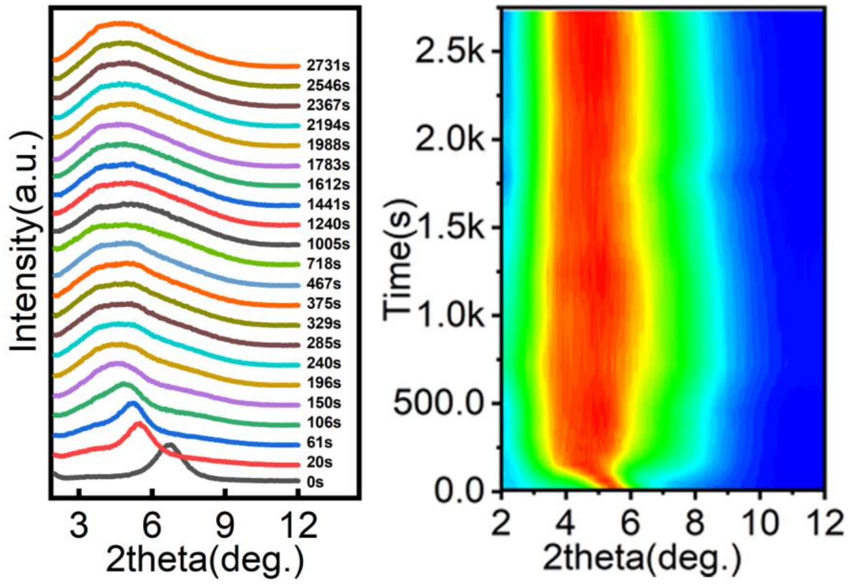

Figure S5. In situ XRD patterns of the immersed MXene film in water.

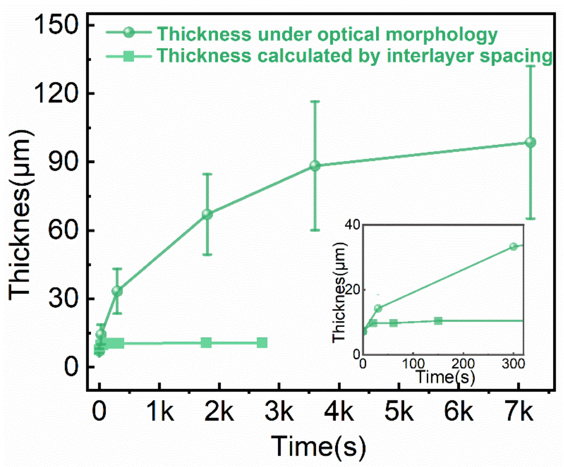

Figure S6. Change in thickness of MXene film during immersion.

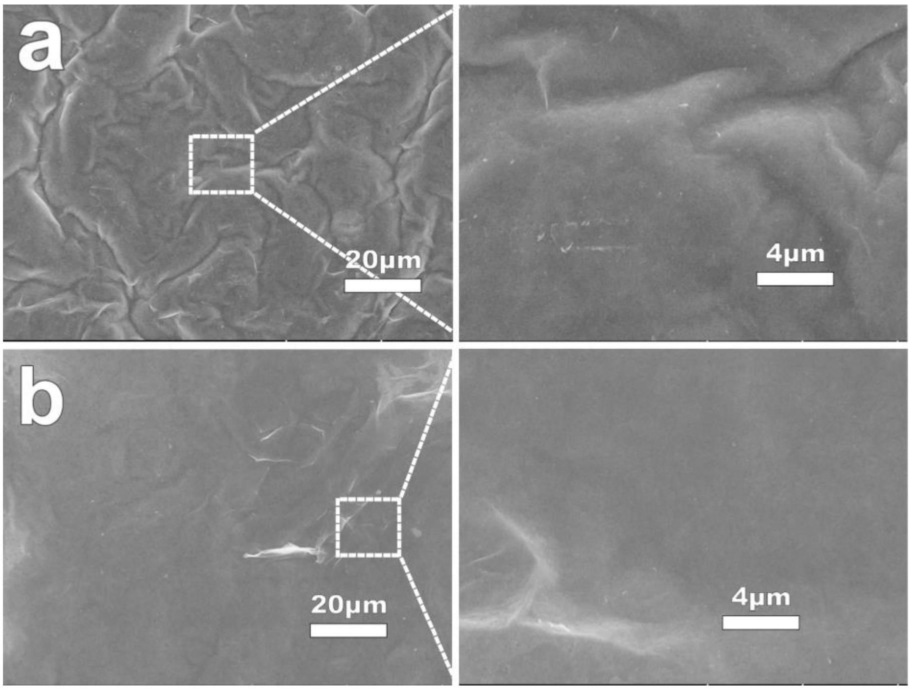

Figure S7. Surface morphology of (a) MXene film and (b) Re-M.

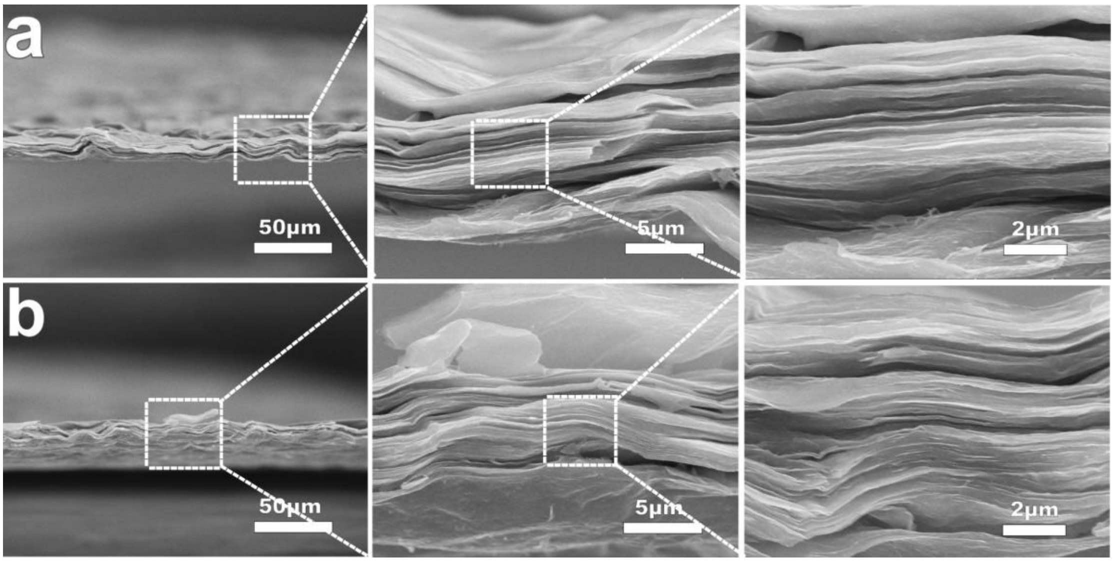

Figure S8. Cross-section morphology of MXene film through cyclic infiltration and drying, (a) 1 th ;(b) 2 th.

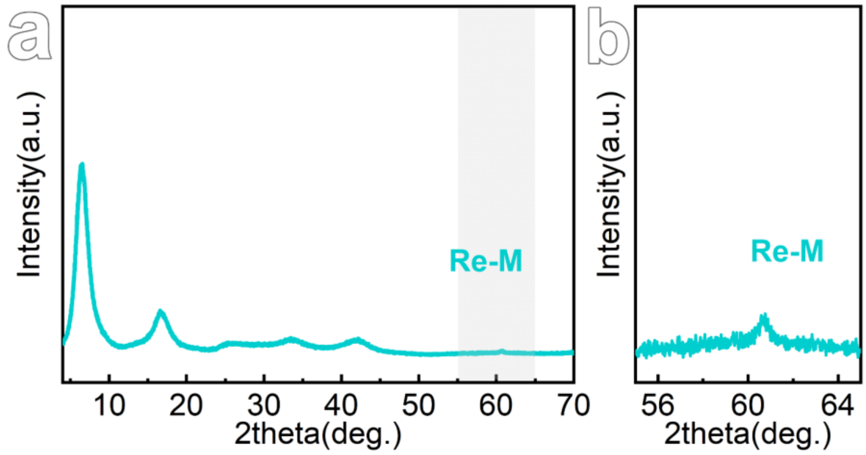

Figure S9. (a) XRD patterns of the Re-M and (b) their detailed XRD patterns around 55°-65°.

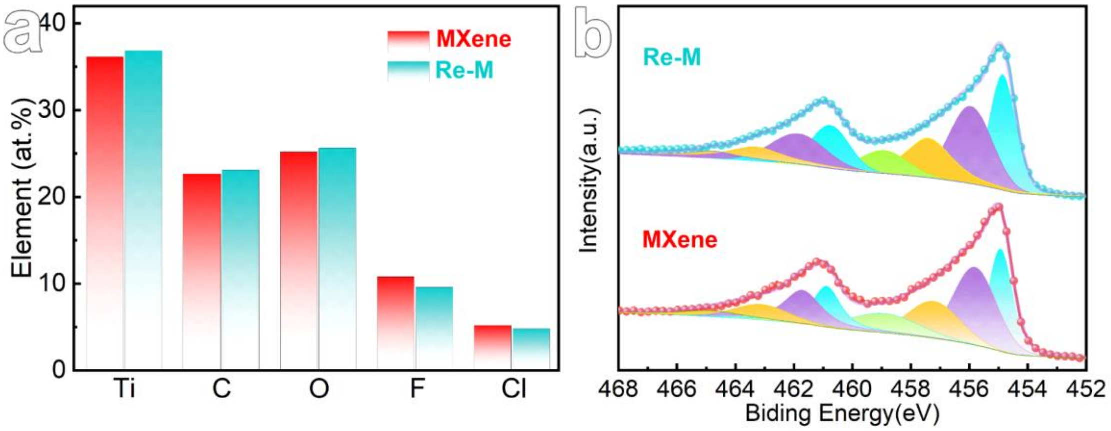

Figure S10. (a) The element content of the MXene film and Re-M; (b) Ti element binding of the MXene film and Re-M.

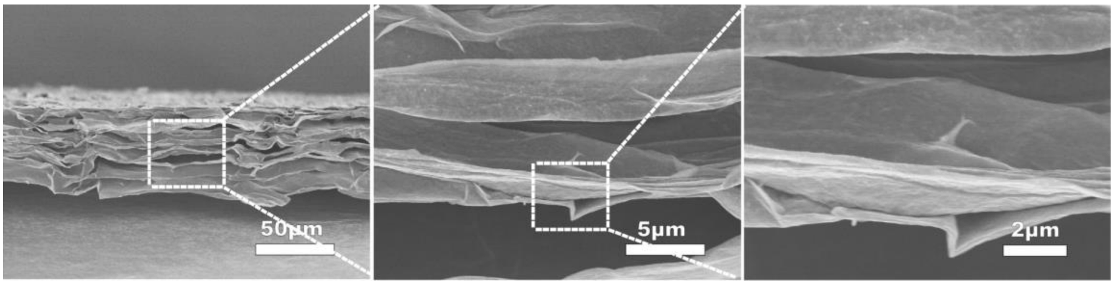

Figure S11. Cross-section morphology of the Re-M after infiltration and drying (named Re-M-S). The preparation of Re-M-S involves Re-M being fully submerged in deionized water and left to stand for 2 h, after which the excess deionized water was removed and dried at 60℃ for 12 h.

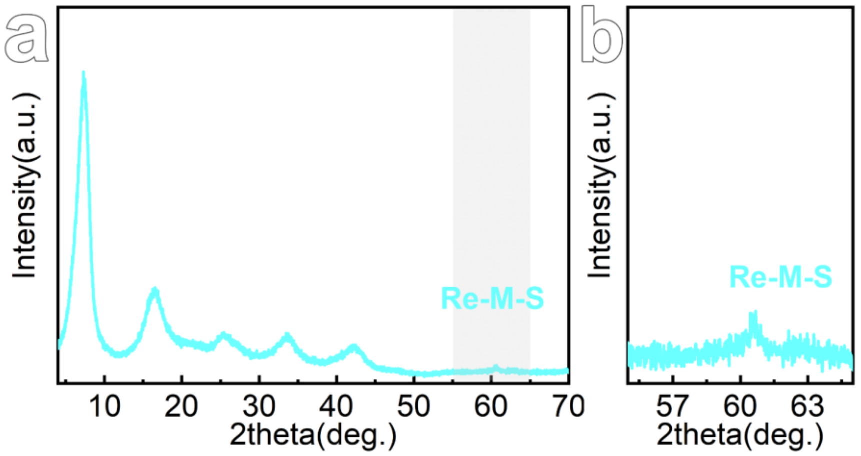

Figure S12. (a) XRD patterns of the Re-M-S and (b) their detailed XRD patterns around 55°-65°.

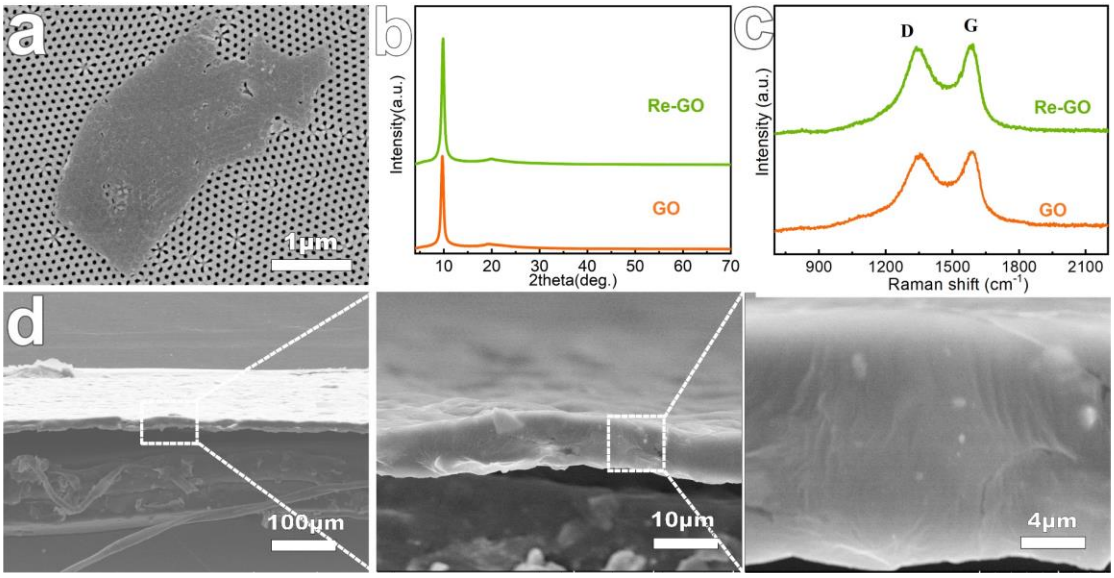

Figure S13. (a) SEM image of GO nanosheet; (b) XRD patterns of the GO film and Re-GO; (c) Raman spectra of GO film and Re-GO; (d) cross-section morphology of the GO film.

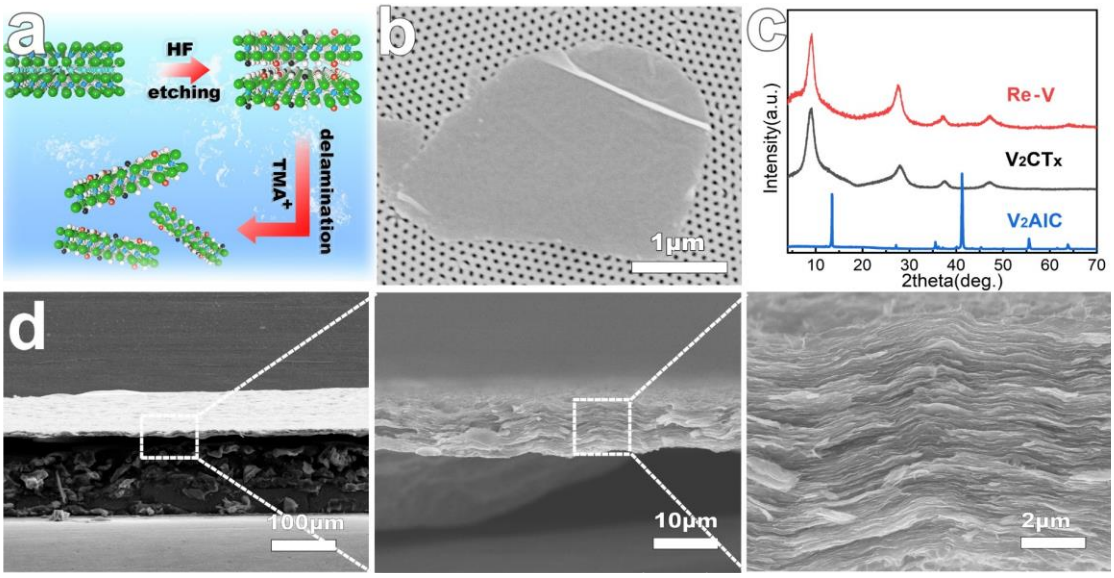

Figure S14. (a) Schematic illustration of the synthesis of V_2CT_x nanosheets; (b) SEM image of V_2CT_x nanosheet;(c) XRD patterns of the V_2AlC, V_2CT_x film and Re-V; (d) cross-section morphology of V_2CT_x film.

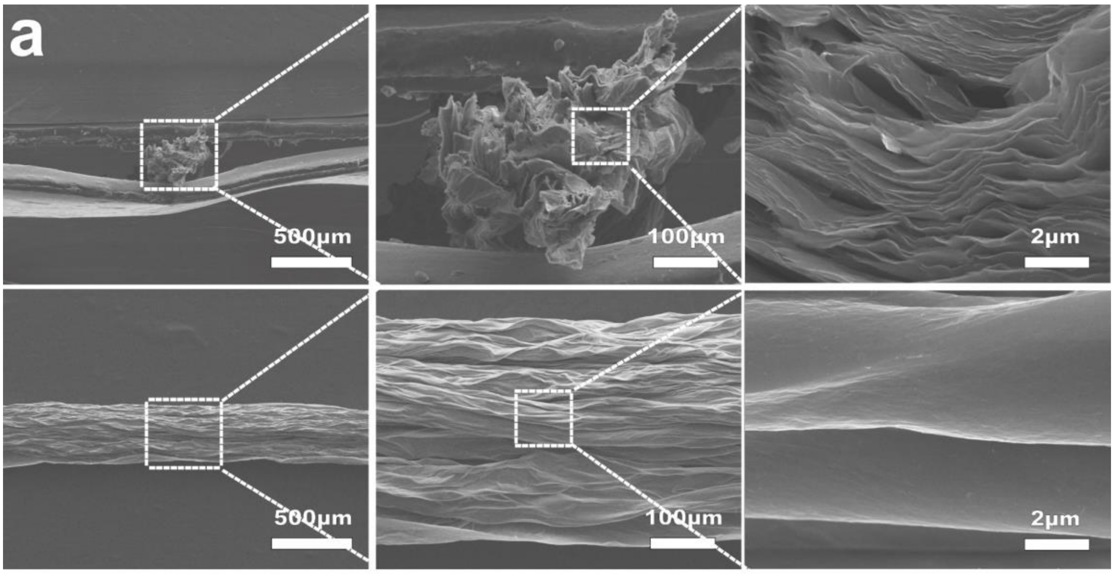

Figure S15. (a) Cross-section morphology of M-fiber; (b) surface morphology of M-fiber.

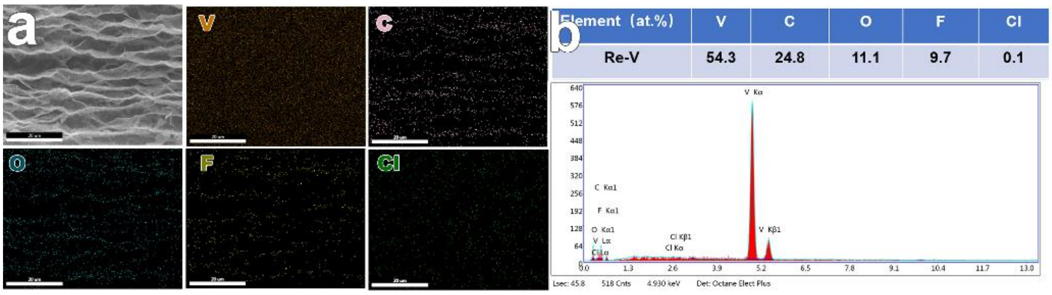

Figure S16. (a) Element distribution of the cross-section of Re-V; (b) Element content of the Re-V.

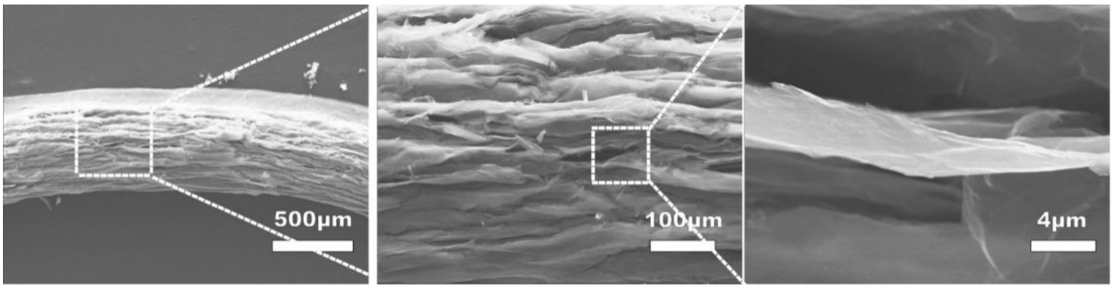

Figure S17. Surface morphology of Re-M-fiber.

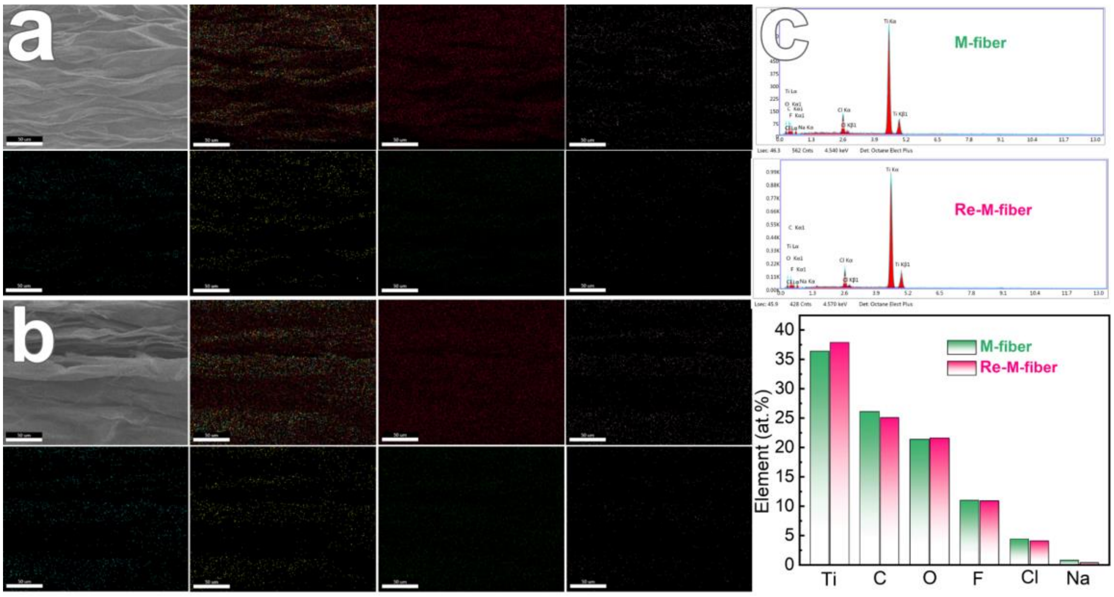

Figure S18. (a) Element distribution of the M-fiber; (b) element distribution of the Re-M-fiber; (c) Element content of the M-fiber and Re-M-fiber.

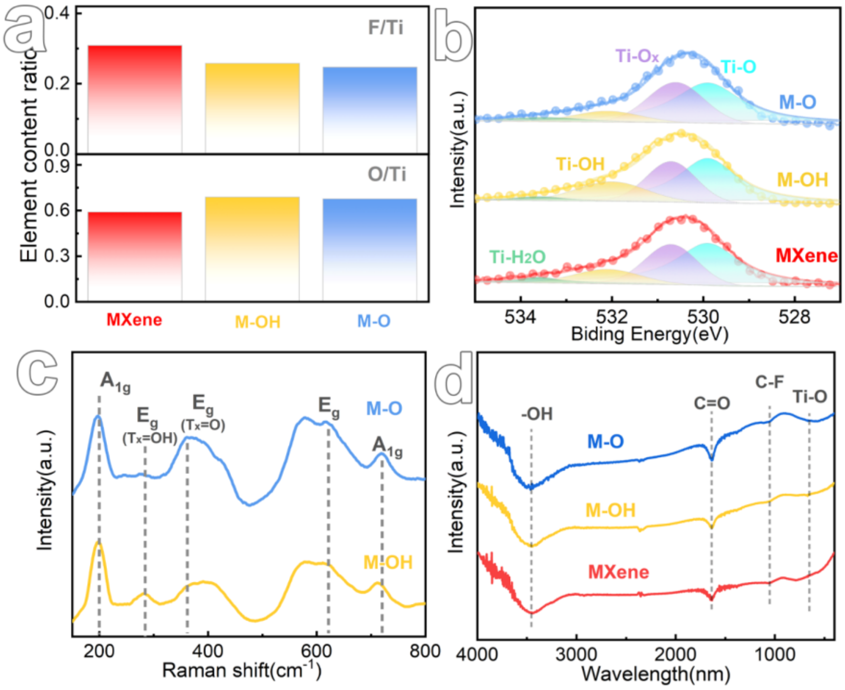

Figure S19. (a) F/Ti and O/Ti ratio of MXene film, M-OH and M-O; (b) O element binding of the MXene film, M-OH and M-O; (c) Raman spectra of M-OH and M-O; (d) FTIR spectra of MXene film, M-OH and M-O.

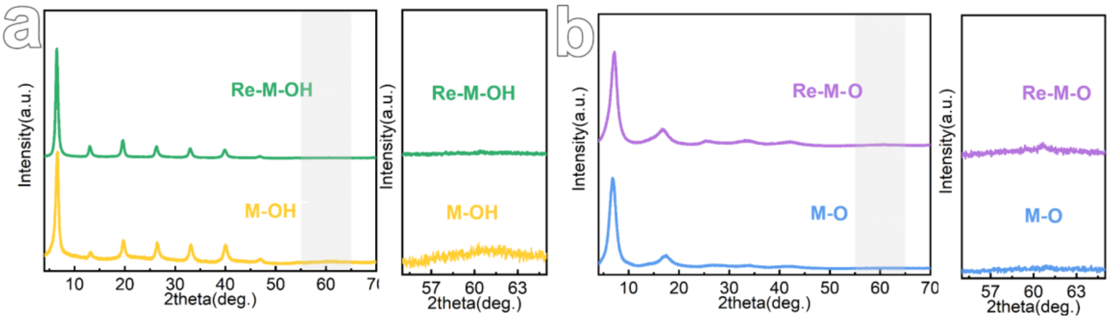

Figure S20. (a) XRD patterns of the M-OH, Re-M-OH and their detailed XRD patterns around 55°-65°; (b) XRD patterns of the M-O, Re-M-O and their detailed XRD patterns around 55°-65°.

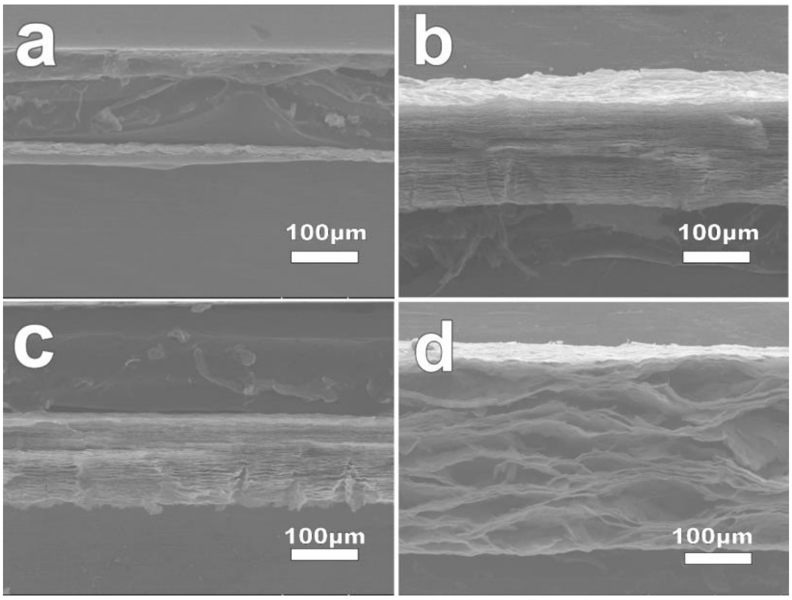

Figure S21. Cross-section morphology of (a) MXene film, (b) Re-M, (c) Re-M-OH and (d) Re-M-O at low magnification.

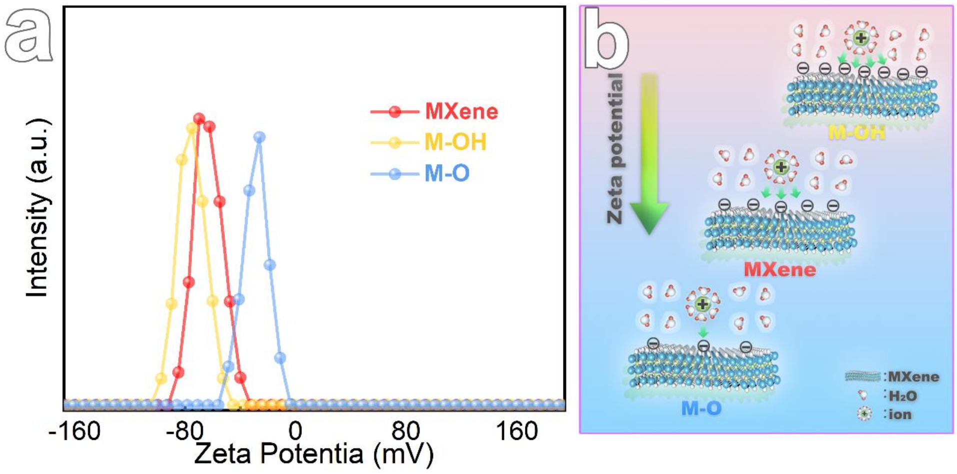

Figure S22. (a) Zeta potential of MXene, M-OH, and M-O. (b) Schematic diagram of electrostatic interaction of MXene, M-OH and M-O nanosheets with interlayer ions.

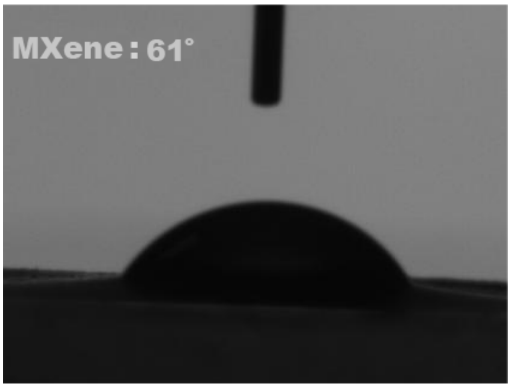

Figure S23. Wetting angle of MXene film.

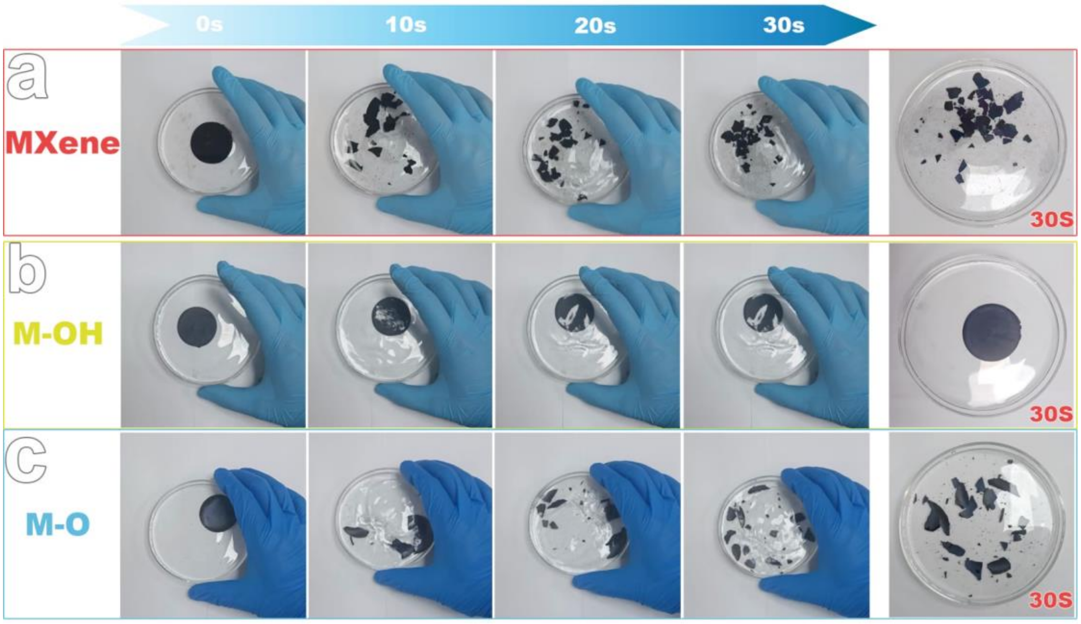

Figure S24. Optical morphology of (a) MXene film, (b) M-OH and (c) M-O after soaking for 2 h and shaking for 30 s with small amplitude in hand.

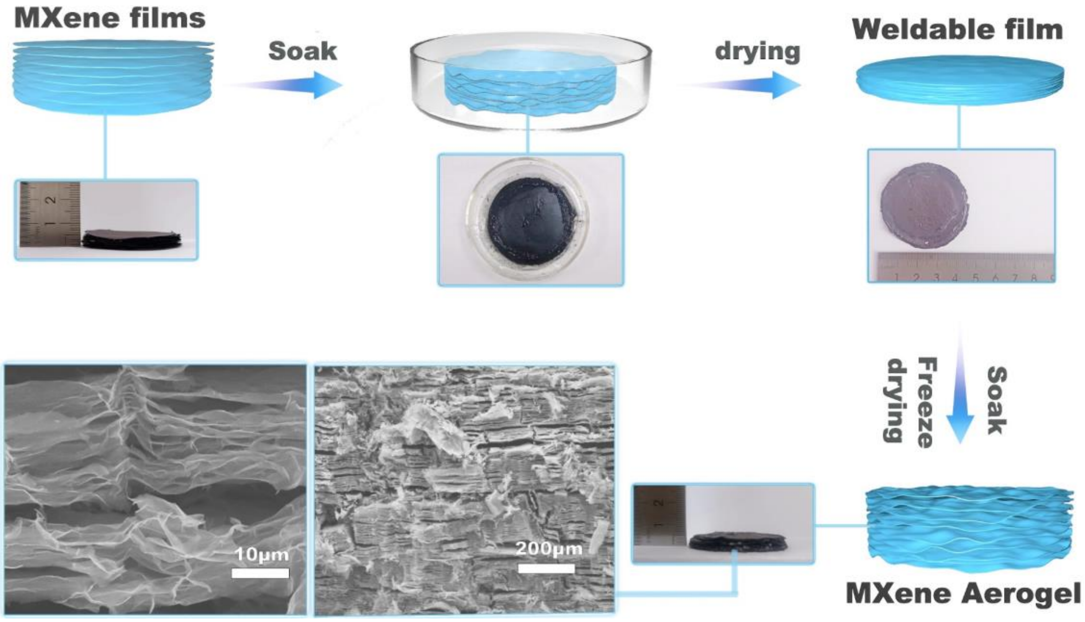

Figure S25. Schematic illustration and cross-section morphology of MXene aerogel prepared by dissociation and reconstruction of the water-assisted welding MXene film. The preparation process is as follows: press 20 MXene films under a pressure of 10 Mpa for 10 min, then place the MXene thick film in deionized water for 1 hour, remove excess deionized water, and dry at 60℃ for 12 h. Then, independently supported aerogels were prepared by referring to Re-M dissociation and reconstruction methods.

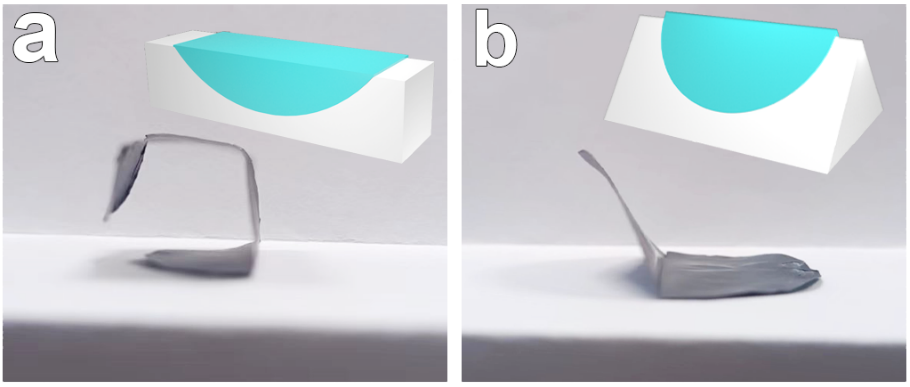

Figure S26. Optical morphologies of three-dimensional porous MXenes films obtained by dissociation and reconstruction attached to different structural surfaces. (a) cuboid; (b) triangular prism.

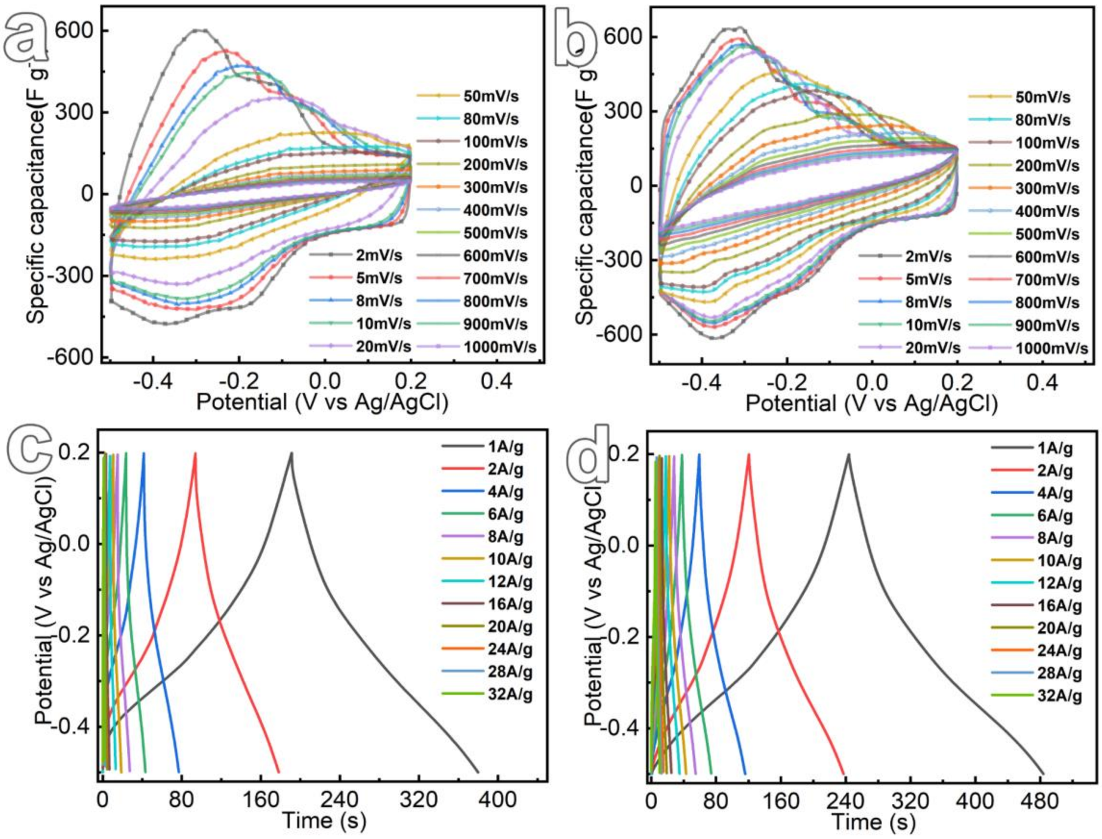

Figure S27. (a) CV curves at different scan rates for MXene film; (b) CV curves at different scan rates for Re-M; (c) GCD curves at different current density for MXene film; (c) GCD curves at different current density for Re-M.

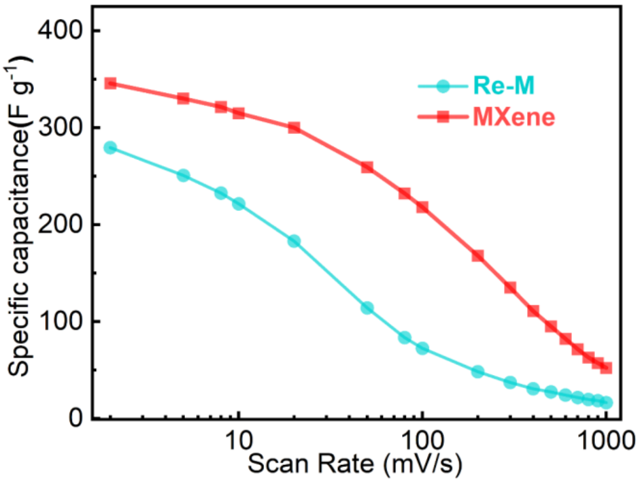

Figure S28. Specific capacitance of MXene film and Re-M at different scan rates.

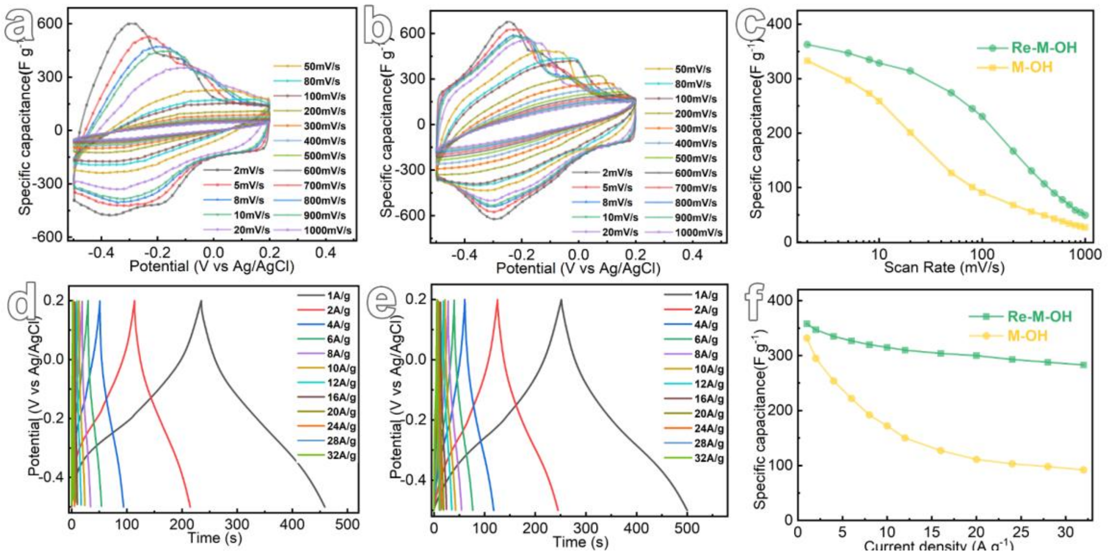

Figure S29. (a) CV curves at different scan rates for M-OH; (b) CV curves at different scan rates for Re-M-OH; (c) specific capacitance of M-OH and Re-M-OH at different scan rates; (d) GCD curves at different current density for M-OH; (e) GCD curves at different current density for Re-M-OH; (f) specific capacitance of M-OH and Re-M-OH at different current density.

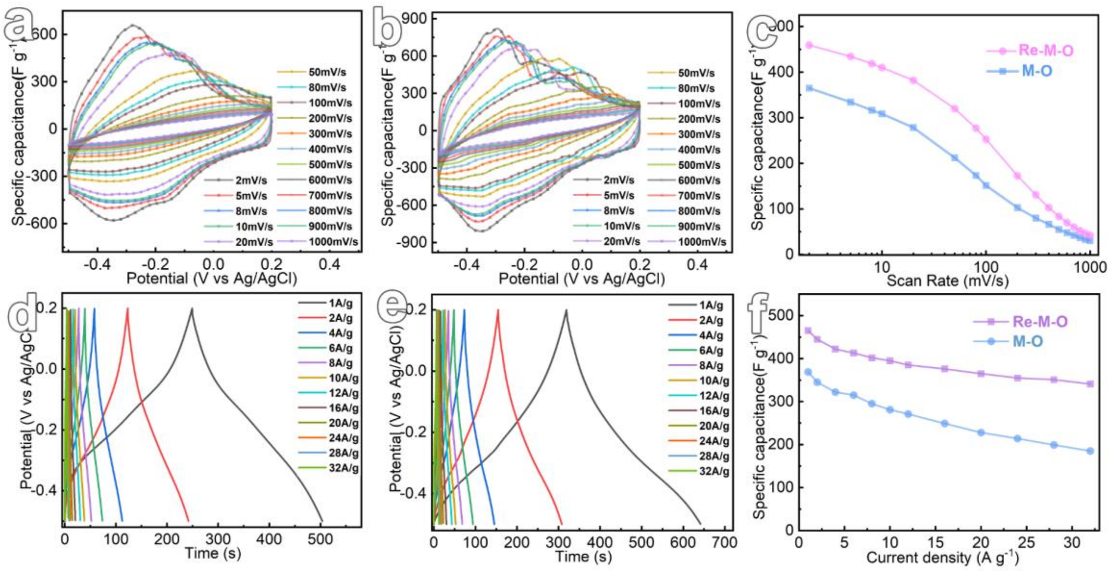

Figure S30. (a) CV curves at different scan rates for M-O; (b) CV curves at different scan rates for Re-M-O; (c) specific capacitance of M-O and Re-M-O at different scan rates; (d) GCD curves at different current density for M-O; (e) GCD curves at different current density for Re-M-O; (f) specific capacitance of M-O and Re-M-O at different current density.

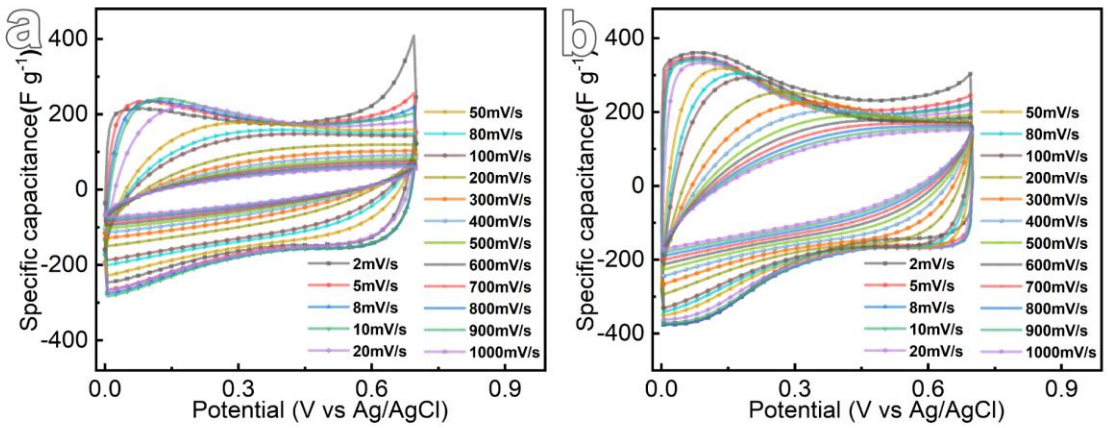

Figure S31. CV curves for (a) MXene film-based and (b) Re-M-based symmetric supercapacitors measured at different scan rates.

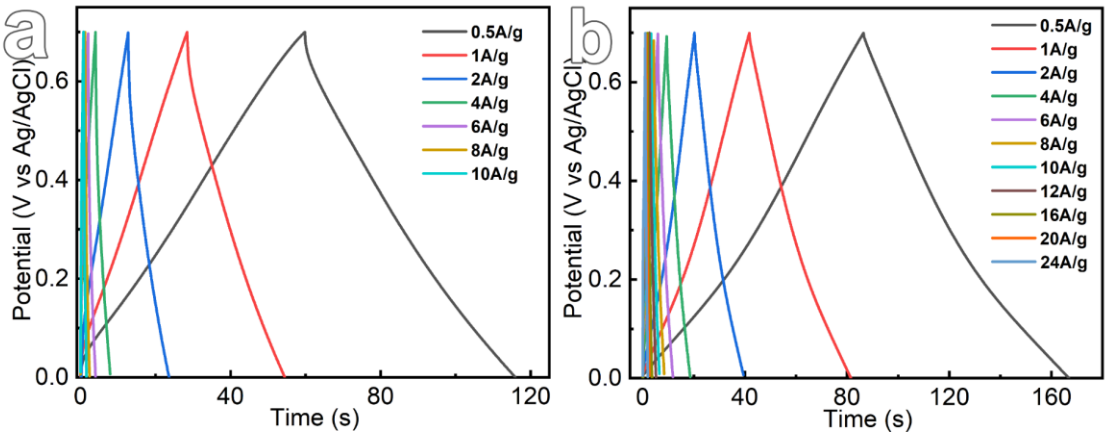

Figure S32. GCD curves for (a) MXene film-based and (b) Re-M-based symmetric supercapacitors measured at different current density.

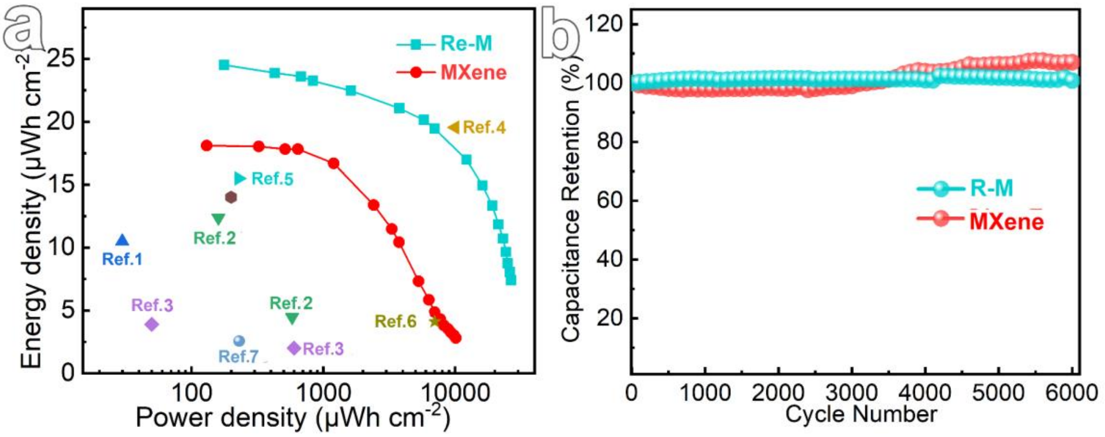

Figure S33. Areal energy and power densities profiles for MXene film and Re-M; (b) corresponding long-term cycle performance measured for 6000 cycles at 200 mV/s.

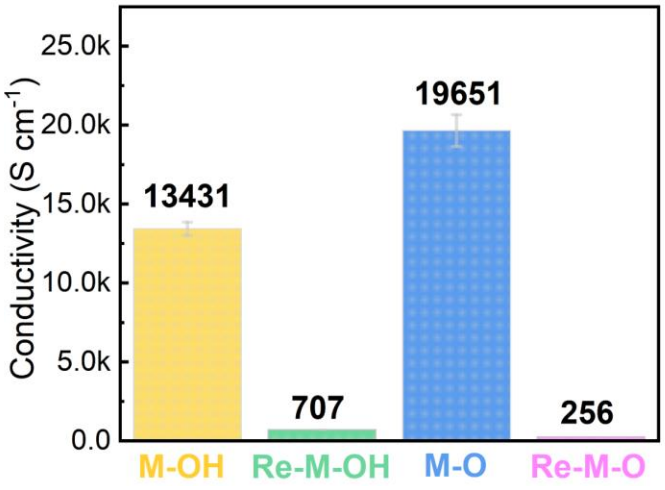

Figure S34. Conductivity of M-OH, Re-M-OH, M-O and Re-M-O.

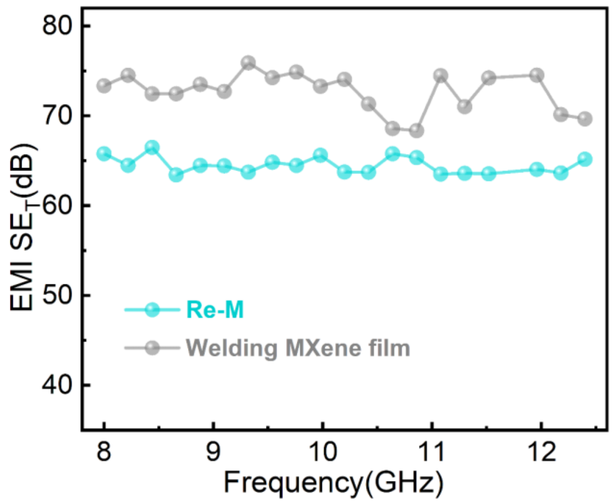

Figure S35. EMI shielding effectiveness of Re-M and water-assisted welding MXene film with a thickness of approximately 150 μm (named Welding MXene film).

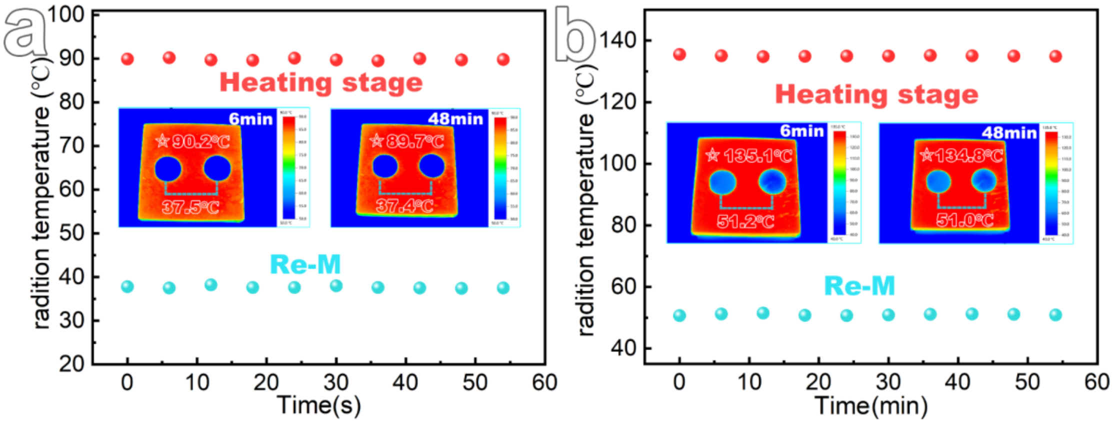

Figure S36. IR stealth performance of Re-M under continuous heating at (a) 45°C and (b) 90°C for 60 min.

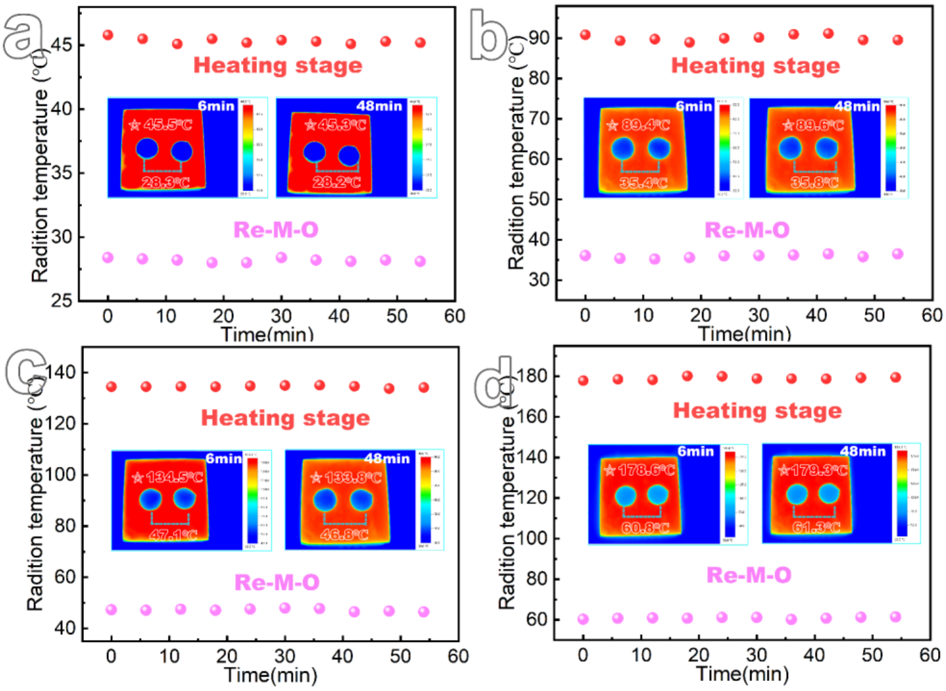

Figure S37. IR stealth performance of Re-M-O under continuous heating at (a) 45°C ,(b) 90°C,(c)135°C and (d) 180°C for 60 min.

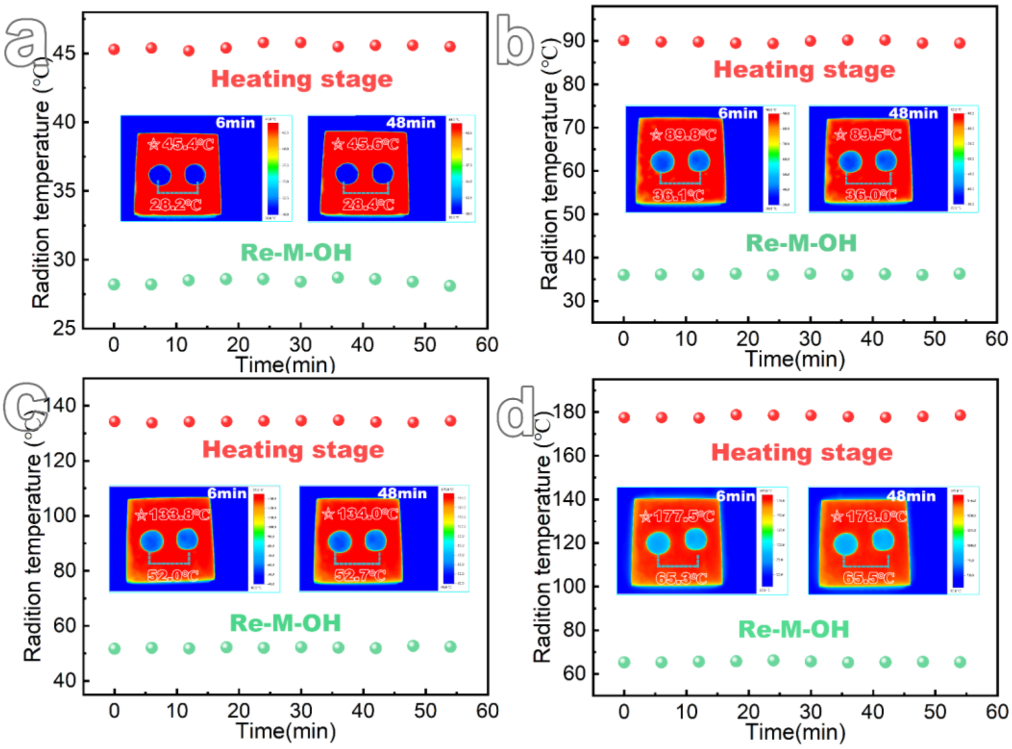

Figure S38. IR stealth performance of Re-M-OH under continuous heating at (a) 45°C, (b) 90°C, (c)135°C and (d) 180°C for 60 min.

Table S1. The electrochemical performance of the restructuring MXenes film compared with other previously reported MXene-based electrodes.

Electrode	Scan rate	Specific capacitance	Current density	Specific capacitance	Electrolyte	Ref.
3D Self-Assembly of MXene Films	2 mV s^{-1}	296 F g^{-1}	-	-	1 M H_2SO_4	8
MXenes Hydrogel	2 mV s^{-1}	272 F g^{-1}	-	-	3 M H_2SO_4	9
Small-flake etched $Ti_3C_2T_x$ film	5 mV s^{-1}	324 F g^{-1}	-	-	3 M H_2SO_4	10
$Ti_3C_2T_x$ -NbN hybrid film	2 mV s^{-1}	277 F g^{-1}			1 M H_2SO_4	11
$Ti_3C_2T_x$ microgels/nanosheets assembly film	10 mV s^{-1}	294 F g^{-1}	-	-	3 M H_2SO_4	12
Vertically aligned MXene film	-	-	1 A g^{-1}	224 F g^{-1}	3 M H_2SO_4	13
3D crumbled MXene	-	-	1 A g^{-1}	333 F g^{-1}	3 M H_2SO_4	14
Holey $Ti_3C_2T_x$ Film	-	-	0.5 A g^{-1}	339 F g^{-1}	3 M H_2SO_4	15
MXene/Graphdiyne nanotube composite films	5 mV s^{-1}	314 F g^{-1}	2 A g^{-1}	337 F g^{-1}	1 M H_2SO_4	16
MXene	2 mV s^{-1}	279 F g^{-1}	1A g^{-1}	274 F g^{-1}	3 M H_2SO_4	This work
Re-M	2 mV s^{-1}	345 F g^{-1}	1 A g^{-1}	346 F g^{-1}	3 M H_2SO_4	This work

Refrence

[1] K. Yang, M. Luo, D. Zhang, C. Liu, Z. Li, L. Wang, W. Chen, X. Zhou, *Chem. Eng. J.* **2022**, *427*, 132002.

[2] S. Uzun, M. Schelling, K. Hantanasirisakul, T. S. Mathis, R. Askeland, G. Dion, Y. Gogotsi, Y. *Small*, **2021**, *17*, 2006376.

[3] H. Tetik, J. Orangi, G. Yang, K. Zhao,S. B. Mujib, G. Singh, M. Beidaghi, D. Lin, D. *Adv. Mater.***2022**, 34, 2104980.

[4] S. A. Kumar, V. Sindhuja, A. Gowdhaman, C. Balaji, R. Ramesh, P. M. Anbarasan, *Electrochim. Acta* **2023**, *459*, 142545.

[5] L.Chi, S. Zheng, J. Ma,Y. Liu,F. F. Xing, F. Zhou,S. Wang, Z. S. Wu, *Carbon* **2022**, *194*, 240-247.

[6] H. Wu, W. Zhang, S. Kandamberth, O. Shekhah, M, Eddaodi, H. N. Alshareef, *Adv. Energy Mater.* **2019**, *9*, 1900482.

[7] Y. Wang, Y. Zhang, J. Liu, G. Wang, F. Pu, A. Ganesh, C. Tang, X. Shi, Y. Qiao, Y. Chen, H. Liu, C. Kong, L. Li, *Energy Storage Mater.* **2020**, *30*, 412-419.

[8] Z. Zhao, S. Wang, F. Wan, Z. Tie, Z. Niu, *Adv. Funct. Mater.* **2021**, *31*, 2101302.

[9] Y. Deng, T. Shang, Z. Wu, Y. Tao, C. Luo, J. Liang, D. Han, R. Lyu, C. Qi, W. Lv, F. Kang, Q.H. Yang, *Adv. Mater.* **2019**, *31*, 1902432.

[10] J. Tang, T. Mathis, X. Zhong, X. Xiao, H. Wang, M. Anayee, F. Pan, B. Xu, Y. Gogotsi, *Adv. Energy Mater.* **2021**, *11*, 2003025.

[11] H. Wang, J. Li, X. Kuai, L. Bu, L. Gao, X. Xiao, Y. Gogotsi, *Adv. Energy Mater.* **2020**, *10*, 2001411.

[12] Z. Wu, X. Liu, T. Shang, Y. Deng, N. Wang, X. Dong, J. Zhao, D. Chen, Y. Tao, Q-H.Yang, *Adv. Funct. Mater.* **2021**, *31*, 2102874.

[13] W. Yang, J.J. Byun, J. Yang, F.P. Moissinac, Y. Peng, G. Tontini, A.W. Dryfe, S. Barg, *Energy Environ. Mater.* **2020**, *3*, 380-388.

[14] X. Zhang, J. Miao, P. Zhang, Q. Zhu, B. Xu, *Chinese Chem. Lett.***2020**, *31*, 2305-2308.

[15] R. Guo, P. Yuan, X. Han, X. He, J. Lu, Q. Li, L. Dang, J. Sun, Z. Liu, Z. Lei, *Small* **2023**, *19*, 2205947.

[16] Y. Wang, N. Chen, Y. Liu, X. Zhou, B. Pu, Y. Qing, M. Zhang, X. Jiang, J. Huang, Q. Tang, B. Zhou,W. Yang, W. *Chem. Eng. J.* **2022**, *450*, 138398.
